# Isocyanide Multicomponent Reactions on Solid Phase: State of the Art and Future Application

**DOI:** 10.3390/ijms21239160

**Published:** 2020-12-01

**Authors:** Naděžda Cankařová, Viktor Krchňák

**Affiliations:** 1 Department of Organic Chemistry, Faculty of Science, Palacký University, 17. listopadu 12, 779 00 Olomouc, Czech Republic; vkrchnak@nd.edu; 2 Department of Chemistry and Biochemistry, 251 Nieuwland Science Center, University of Notre Dame, Notre Dame, IN 46556, USA

**Keywords:** convertible isocyanides, drug discovery, multicomponent reactions, post-Ugi transformations, solid-phase synthesis, Ugi four-component reaction

## Abstract

Drug discovery efforts largely depend on access to structural diversity. Multicomponent reactions allow for time-efficient chemical transformations and provide advanced intermediates with three or four points of diversification for further expansion to a structural variety of organic molecules. This review is aimed at solid-phase syntheses of small molecules involving isocyanide-based multicomponent reactions. The majority of all reported syntheses employ the Ugi four-component reaction. The review also covers the Passerini and Groebke-Blackburn-Bienaymé reactions. To date, the main advantages of the solid-phase approach are the ability to prepare chemical libraries intended for biological screening and elimination of the isocyanide odor. However, the potential of multicomponent reactions has not been fully exploited. The unexplored avenues of these reactions, including chiral frameworks, DNA-encoded libraries, eco-friendly synthesis, and chiral auxiliary reactions, are briefly outlined.

## 1. Introduction

Multicomponent reactions (MCRs) are one-pot highly efficient transformations utilizing three or more components capable of creating highly diverse compounds. They are also atom-efficient, incorporate a substantial portion of components, and do not require any additional reagents. MCRs are ideal tools for the construction of vast arrays of biologically relevant molecules [1].

One of the most explored and the most frequently described MCRs is the Ugi four-component reaction (U-4CR), often named Ugi four-component condensation (U-4CC). The U-4CC enables the formation of a functionally complex organic molecule with a single synthetic transformation in comparison to numerous steps applied in conventional methodologies [2]. An illustrative example to generate structurally complex compounds resulted from Schreiber’s laboratory [3]. In addition, MCRs are also attractive for automated parallel synthesis [4].

A large number of MCRs performed in solution phase have been reported, while solid-phase applications appear less frequently. In this review, we include publications using isocyanide as one of the reaction components (so-called isocyanide-based multicomponent reactions, IMCRs). The majority of the presented syntheses utilized the Ugi reaction either as a one-step process resulting in the target products (not considering the additional step of cleavage from the resin) or as one of the steps in a several-step synthesis, which involves a transformation of the primary product. We also included Ugi four-center three-component reactions (U-4C-3CRs) as well as nontraditional Ugi reactions, e.g., Ugi-azide four-component reactions [5,6], or transformations unrelated to the Ugi reaction [7,8,9]. To the best of our knowledge, there are only two examples of Passerini reactions leading to small molecules via a solid-phase reaction [10,11].

Initially employed for the formation of dipeptides, the Ugi reaction has been applied for the synthesis of complex structures [3] or natural products [12], and currently, it plays an important role for finding biologically relevant molecules [13]. Numerous research groups worldwide have contributed to this ground-breaking one-pot reaction. MCRs are very attractive due to their potential and interdisciplinary applications. MCRs are advanced tools for sustainable organic synthesis [14] due to their good compatibility with green solvents [15], high atom economy, efficiency, and mild reaction conditions. At present, the solid-phase Ugi reaction is also essential in the synthesis of macropeptides, glycopeptides, lipoproteins, and in the field of ligation and bioconjugation [16,17]. Furthermore, IMCRs are attractive for their compatibility with DNA-encoded combinatorial synthesis [18]. In addition, chiral auxiliary has already been shown to enable direct enantio-enriched compounds [19,20]. MCRs also have the potential to provide a basis for large-scale molecular data storage [21].

This review is focused on the synthesis of small molecules on solid supports and demonstrates the various methodologies and strategies used in IMCRs. Herein, the presented approaches are classified according to the type of IMCRs and taking into account whether the IMCR was the only chemical transformation leading to the target products or whether it was one of several steps in the synthetic sequence. In certain sections, the reactions are classified according to the functionality bound to the solid support.

## 2. Isocyanide-Based Multicomponent Reactions (IMCRs)

The discovery and course of IMCRs is explained in detail and comprehensively illustrated by Ugi and Dömling [22,23]. Briefly, isocyanides were discovered in 1859 by Lieke, who prepared them by the random reaction of allyl iodide and silver cyanide [24]. The term “isonitrile” was introduced by Gautier and simultaneously by Hoffmann, who prepared these molecules by reacting primary amines with chloroform and alkali [25]. Note that we used the term “isocyanide” within this text because it is preferred by IUPAC (International Union of Pure and Applied Chemistry).

**Scheme 1:**
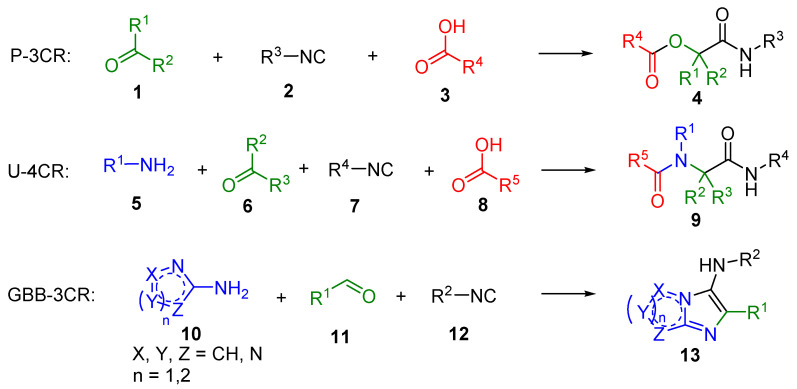
Types of isocyanide-based multicomponent reactions (IMCRs) described in this review.

Approximately a half-century later, in 1921, Passerini introduced the first MCRs of isocyanides. Passerini three-component reaction (P-3CR) of a carbonyl compound, isocyanide, and carboxylic acid afforded α-acyloxy carboxamide **4** (Scheme 1). Since the 1950s, attempts to prepare isocyanides by a more convenient method have appeared in the literature [26]. Finally, the beginning of U-4CR dates to 1959, when Ivar Karl Ugi (1930–2005) performed a one-pot reaction of amine, carbonyl compound, isocyanide, and acid, leading to α-acylamido carboxamide **9 [7,27,28]**. Since 1962, the reaction is referred to as the Ugi reaction (U-4CR). Approximately twenty years later, the transformation was also performed on a solid phase [29]. The first stereoselective Ugi reaction on the solid phase was accomplished in 2000 by Kunz et al. [19]. Finally, the newest MCR is the Groebke-Blackburn-Bienaymé three-component reaction (GBB-3CR, product **13**) [30,31,32].

### 2.1. Practical Assessments

**Scheme 2:**
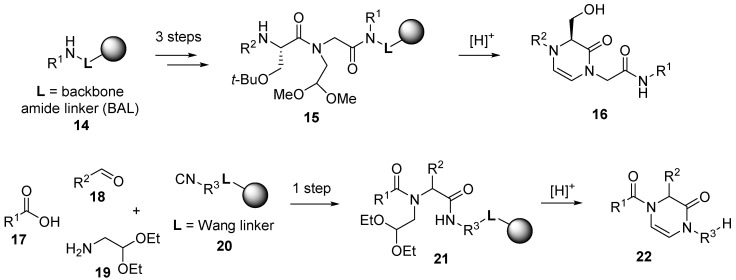
Comparison of classical synthesis and Ugi four-component reaction (U-4CR).

From a practical point of view, a single synthetic transformation instead of traditional follow-up synthesis is one of the advantages of IMCRs, which is reflected in the large interest in MCRs. The feasibility of the synthesis involving the Ugi reaction in comparison to the standard stepwise synthetic protocol is illustrated in the following example (Scheme 2).

While the conventional synthesis of linear precursor **15** involved 3 steps [33], analogous intermediate **21** was formed by U-4CR in only one step [34]. Acidic treatment initiated a cleavage of the protecting groups as well as simultaneous cleavage of the intermediate from the resin and cyclization to the target 3,4-dihydropyrazin-2(1*H*)-one **16** and **22**.

A reduction in the synthetic steps is one of several advantages of IMCRs. Other advantages are high atom-efficiency (when only one molecule of water is lost) and usually high yields of product. The yields of the products are primarily sensitive to the nature of the aldehyde [35]. The aliphatic aldehydes and aromatic aldehydes bearing electron-donating groups (EDGs) afforded good yields in comparison to the aldehydes containing electron-withdrawing groups (EWGs, e.g., References [35,36,37,38]). Even larger excess reagents or repeated addition of fresh reagents often did not improve the yield [35,38], although the opposite observation was also described [39]. In addition to the poor reactivity of aromatic aldehydes in the Ugi reaction, aromatic isocyanides were also reported to be less reactive [40].

Since a limited number of commercially available isocyanides greatly reduces the structural diversity of a library [35], convertible isocyanides play an important role in the achievement of diverse products. Another important aspect of IMCRs is the unpleasant odor of most isocyanides. This odor can be circumvented by immobilization of isocyanides onto a polymer support, which is an advantage of solid-phase synthesis (SPS) over solution-phase synthesis. The other option to avoid the odor is the preparation of the isocyanide in situ, which has been applied so far only in the solution phase [41,42].

The scope of the reaction components is not limited to amine, aldehyde, carboxylic acid, and isocyanide—alternative reagents were successfully examined. For example, sulfonamide was used as an amine reagent [43], imine was directly bound to the Rink resin [44], or ketones were applied to replace the aldehydes despite their lower reactivity [45,46,47,48,49]. Alternatively, glyoxal was used as the aldehyde component in U-4CR, and the second carbonyl functionality was employed for the imidazole ring formation [50]. Alcohol was used as an acid component [39], or application of thiocarboxylic [51,52], cyanic [53,54], thiocyanic, or hydrazoic acid [54] was reported in the literature. Alternatively, one starting component can bear two functional groups, e.g., ketoacid, thus serving as both ketone and carboxylic acid [1,54]. A list of resin-bound starting materials of IMCRs was shown by Dömling [55].

Regarding the solvents, the Ugi reaction is typically performed in methanol (MeOH) in the case of solution-phase synthesis. In solid-phase chemistry, it is essential to consider the swelling properties of the respective resin. Typical SPS is carried out using hydrophobic polystyrene (PS)-based resin, which shrinks in MeOH. Therefore, mixtures of solvents are used, including MeOH/tetrahydrofuran (THF) and MeOH/dichloromethane (DCM) in various ratios (e.g., References [3,35,56,57,58]), while the usage of MeOH/CHCl_3_ [36,59], MeOH/CHCl_3_/H_2_O [53,54,60], or DCM/DMF [37] was reported much less frequently. In addition, polar TentaGel resin, composed of PEG-grafted PS, was successfully used in MCR [37,39,46,60]. U-4CR was even successful when H_2_O was used (in the case of filter paper used as solid support) [38,61,62], and 2-methyltetrahydrofuran (2-MeTHF) [15] was described recently as a green solvent in the Ugi reaction. All practical aspects of U-4CC, performed in solution phase, were discussed in detail [63].

To be objective, the Ugi transformation suffers from a few shortcomings, which, however, cannot outperform the overall MCR benefits. The first shortcoming is the long reaction times needed (up to several days); however, the reaction time can be significantly shortened (to ≤5 min) by using microwave-irradiation [37]. The longer reaction times are also connected with the nature of the solid support, and PS resin suffers from slow reaction kinetics [64]. Furthermore, the stereochemical outcome is highly dependent on the chirality of the reagents. It is known that chiral aldehydes have the greatest effect on the diastereoselectivity due to the proximity to the newly formed stereocenter [57]. Amino-carbohydrates were used as an immobilized chiral auxiliary [19,20]. Asymmetric MCRs were reviewed by Ramón and Yus [65].

Concerning the solid-phase arrangement, this approach offers great advantages over the solution-phase chemistry. The most apparent benefits are facilitated washing of the reagents and isolating of the target products, and the ability to carry out parallel syntheses to produce large libraries containing thousands of compounds intended for biological screening. The practical aspects of combinatorial solid-phase organic synthesis were summarized [66].

### 2.2. Conventions

**Figure 1:**
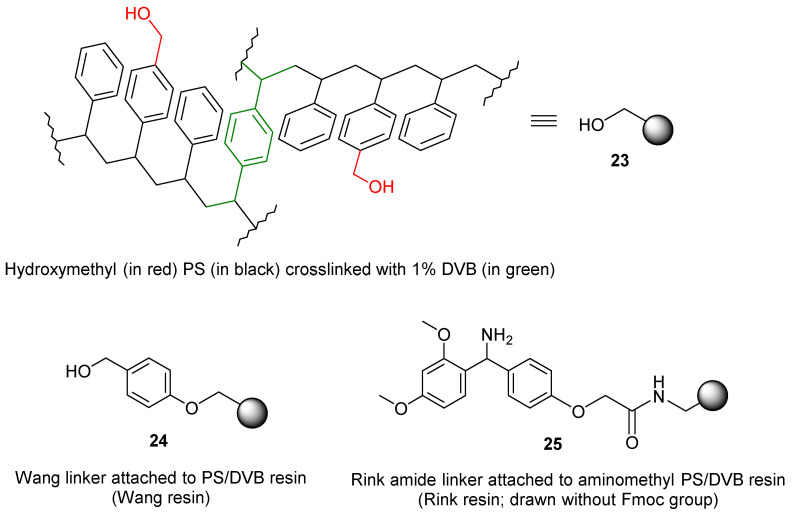
Solid-phase supports most frequently used in the review.

The most frequently used resin for SPS is PS crosslinked with 1% divinylbenzene (DVB, **23**, Figure 1). This support is depicted in all schemes and figures by a solid ball. A chemical entity, including linkers, is drawn from the ball and is assumed to be attached predominantly to the para-position of the aromatic ring. A linker, abbreviated with the letter “L”, is always specified. The structures of the two most common linkers, Wang (**24**) [67], and Rink amide linkers (**25**) [68], are shown in Figure 1. The Rink linker can be attached to the resin via amide (shown) or ester bonds.

## 3. Resins

To carry out the U-4CC on a solid phase, one component needs to be attached to the insoluble resin support. The resin contains a linker allowing for immobilization of the selected component and enabling the release of the product once the SPS is accomplished. The solid-supported component can be assembled in solution and then attached to a resin, or it can be assembled on the resin. Any of the four components, amine, aldehyde, carboxylic acid, or isocyanide, can be immobilized for U-4CC; however, the most frequently used components are amine and isocyanide.

### 3.1. Amine Resin

**Scheme 3:**
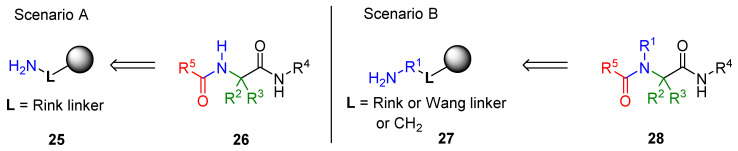
Immobilization of the amino component.

The immobilization of the amino component offers two different synthetic scenarios (Scheme 3). The ammonia equivalent is attached directly to a linker; in this scenario, the Ugi product, after cleavage from the resin, does not have a substituent on the amide nitrogen (**26**, Scenario A). The other option is to attach the amino group to the resin via a moiety R^1^ (**27**, Scenario B). In the latter case, the product has four diversity positions, and the amide is substituted by the R^1^ group.

In general, the amino group containing resin (Rink resin, solid supports modified by amino acids or any other components bearing primary amino groups) serves as an ammonia equivalent. The use of ammonia in the solution phase often leads to the formation of a significant number of unidentified products [69]. The solid-phase approach offers a solution to this issue.

Typical resin for Scenario A (Scheme 3) is commercially available Rink amide resin **25**, which provides products bearing secondary amides [15,35,37,57,58,69,70,71]. There is no structural limit on resin-bound amino derivatives for carrying out the synthesis according to Scenario B. Wang and hydroxymethyl resins esterified with amino acids were frequently used [4,36,46,62], as well as Rink and aminomethyl resins acylated with amino acids [45]. Other examples include heterocycles, nucleosides [72], and carbohydrates [19,20].

### 3.2. Isocyanide Resin

**Scheme 4:**
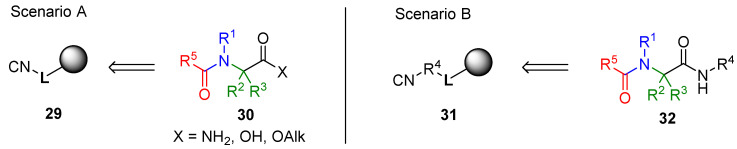
Formation of the resin-bound isocyanides.

Unlike amines, carboxylic acids, and aldehydes, structurally varied isocyanides are not commercially available. The synthesis of isocyanides suffers from several drawbacks, including multistep synthesis and unpleasant odor. However, the polymer-supported isocyanides are completely odor-free, which is an advantage of the IMCRs performed on the solid phase. Two different strategies for the preparation of isocyanide resins were developed: (i) the isocyanide-containing reagent was prepared in solution and then attached to the resin, or (ii) the isocyanide was formed on the resin, typically from a resin-bound amine by formylation followed by dehydration [8,29,50]. Analogous to the amine resins, the isocyanide was either directly linked to a resin-bound linker (typically Rink amide linker, **29**, Scenario A, Scheme 4) or a diversity element R^4^ was introduced between the isocyanide and the linker (**31**, Scenario B) [73]. Accordingly, three or four diversity position products were formed.

**Scheme 5:**
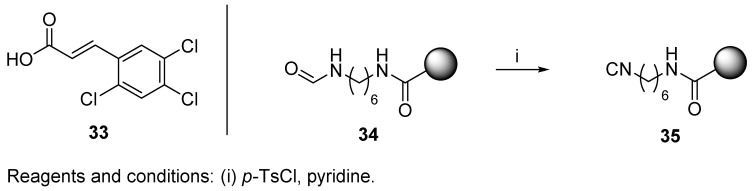
Resin-bound isocyanide on a dedicated polymer support.

First, we discuss the most common route to polymer-supported isocyanide by dehydration of formyl amides. In 1981, isocyanide resin in U-4CC was reported for the first time by Arshady and Ugi [29]. Their original work [29] focused on peptide synthesis by ligation of a C-terminal fragment with an N-terminal fragment. Dedicated PS and polyacrylamide polymers crosslinked with bisacrylamide were prepared and evaluated. Copolymerization included 2,4,5-trichlorophenylacrylate **33** (Scheme 5), which introduced a functional group for subsequent reaction with diamine and formylation (resins **34**). Both formamide polymers were subsequently dehydrated with an excess of *p*-TsCl in pyridine (resins **35**) and afforded comparable results. The authors selected the polar polyacrylamide polymer to carry out the scope and limitation experiments described in Section 4.1.2.

**Scheme 6:**
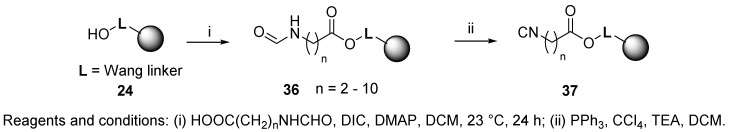
Preparation of Wang resin-bound isocyanide.

On-resin dehydration was used very frequently. Mjalli et al. reported the preparation of Wang resin-bound isocyanide **37** (Scheme 6) and its usage for the synthesis of imidazoles (cf. Scheme 57) [50]. It was further utilized for syntheses of other heterocycles, such as 2-thiohydantoin 4-imides, hydantoin 4-imides, tetrazoles, and lactams (cf. Section 8.2 and Section 9.2.2) [53,54]. The synthetic strategy was based on formylation and dehydration. Formylated resin-bound amino acids, prepared by reaction with HCOOEt or HCOOH in the presence of Ac_2_O, were attached to Wang resin **24**, and precursor **36** was subjected to dehydration with PPh_3_ and CCl_4_ in DCM in the presence of triethylamine (TEA) to produce target resin **37**. The resin was reportedly stable for up to 6 months at 23 °C, when kept in a vacuum desiccator. However, the usage of carcinogenic CCl_4_ makes this method less desirable.

The synthesis of isocyanides was also evaluated on various resins, including aminomethyl PS, TentaGel-NH_2_, polydimethylacrylamide, copolystyrene-acrylate, copolystyrene-acrylamide, and copolydimethylacrylamide-acrylate, via formylation with HCOOH/Ac_2_O and dehydration with TsCl in pyridine [74].

**Scheme 7:**
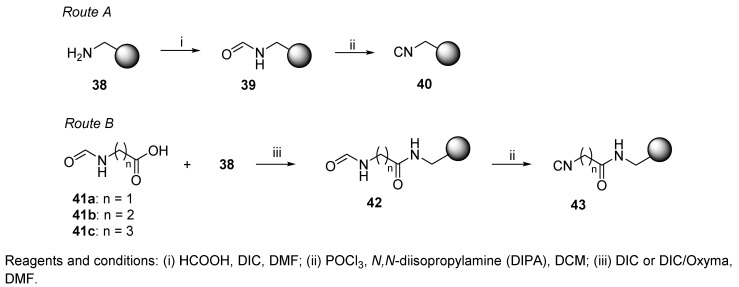
Preparation of aminomethyl resin-bound isocyanide.

Less hazardous dehydration conditions were applied by another research group [49]. Inexpensive aminomethyl PS resin was functionalized with an isocyano group, which was either directly generated on the resin (**40**, Scheme 7) or attached via a spacer (**43**). The spacer unit (Gly, β-Ala, γ-aminobutyric acid (GABA)) decreased the steric hindrance at the isocyanide functional group and thereby facilitated the Ugi reaction. The preparation of both isocyanide resins is outlined in Scheme 7 (Route A, Route B). Oxyma (ethyl cyano(hydroxyimino)acetate) was added to facilitate the coupling reaction of formylated β-Ala **41b** and GABA **41c** with aminomethyl resin **38**. Formylated amino acids **41a**–**41c** were prepared by reaction of amino acid with HCOOH in the presence of acetic anhydride. While the reaction worked well for Gly and β-Ala (**41a** and **41b**), the formylation of GABA afforded a lower yield of 41c due to internal cyclization and formation of pyrrolidin-2-one. Regarding the stability of the resins, the authors reported that formylated resins **42** were stable for months; in contrast, isocyanide resins **43** decomposed in a few weeks. The resins were used for the synthesis of α,α-dialkylglycines as highly hindered C-tetrasubstituted amino acids (cf. Scheme 27).

**Scheme 8:**
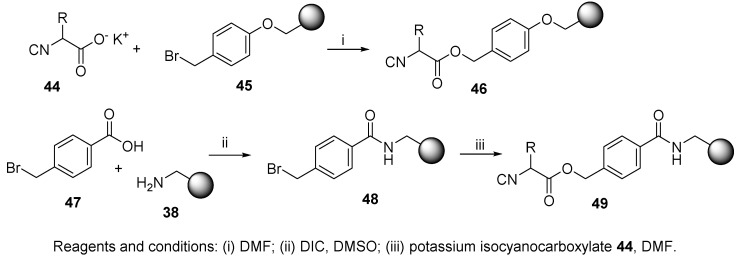
Preparation of isocyanide resins from brominated resins and potassium isocyanocarboxylates.

The next example shows the immobilization of isocyanide prepared in solution. This new method for the synthesis of resin-bound isocyanocarboxylic acids was described by Henkel and coworkers (Scheme 8) [73]. They developed a direct coupling method of four different potassium isocyanocarboxylates **44** (prepared from the corresponding acids by ethanolic KOH) onto two brominated resins **45** and **48**. The ester bond cleavage of the Ugi product with 3-methylbutylamine yielded amides, and treatment with LiOH provided acids (cf. Scheme 33).

**Figure 2:**
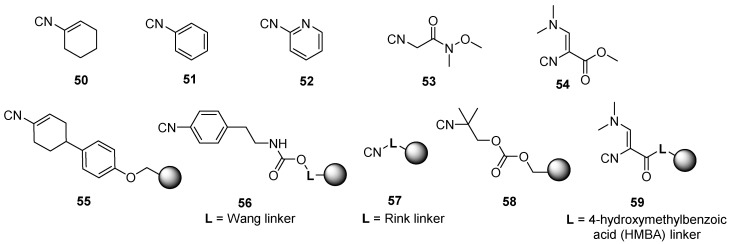
Solution-phase and resin-bound convertible isocyanides.

The lack of commercially available isocyanides prompted the design and synthesis of dedicated linkers for the attachment of isocyanide to the resin. These resins are referred to in the literature as universal (also named versatile or convertible) isocyanide resins and enable post-Ugi conversion of the amide to other functional groups. They allow for the release of the Ugi products from the resin through cleavage of the secondary amide bond and yielding the final products as amides, acids, or esters. The first reported convertible isocyanide was 1-isocyanocyclohexene **50** (Figure 2), which was discovered by Ugi and Rosendahl in 1963 [75], and referred to as Armstrong isocyanide [76,77]. Other convertible isocyanides are isocyanobenzene **51**, 2-isocyanopyridine **52**, Weinreb-type amide **53**, and 3-*N*,*N*-(dimethylamino)-2-isocyanoacrylate **54**. Regarding the resin-bound, cyclohexenyl isocyanide resin (Armstrong-type isocyanide) **55**, safety-catch linker isocyanide resin **56**, universal Rink isocyanide resin **57**, carbonate convertible isocyanide (CCI) resin **58**, and 3-*N*,*N*-(dimethylamino)-2-isocyanoacrylate resin **59** were developed for MCRs. The application of those isocyanides is covered in Section 6 and Section 9.2.1.

**Scheme 9:**
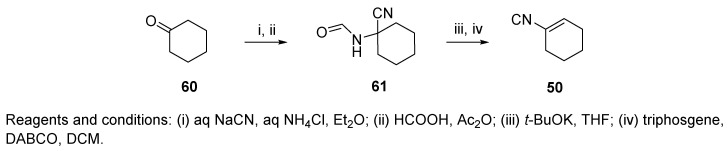
Preparation of Armstrong isocyanide.

The Armstrong cyclohexenyl isocyanide **50** was originally prepared from *N*-(1-cyanocyclohexyl)formamide **61** by dehydration with phosphorus oxychloride by Rosendahl and Ugi [75]. Subsequently, the yields were improved by using a different dehydrating agent, triphosgene, and by purifying **61** prior to dehydration (Scheme 9) [78]. The Armstrong-type isocyanide was also prepared on the solid phase by Piscopio and coworkers (resin **55**, Figure 2) [79].

**Scheme 10:**
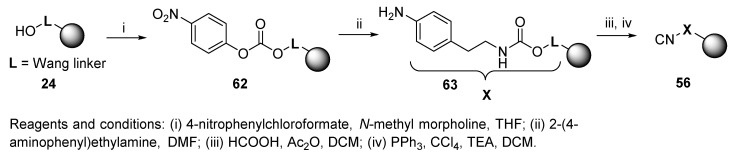
Synthesis of safety-catch linker.

Convertible safety-catch linker **56** was prepared from resin-bound aniline **63** via formylation and subsequent dehydration (Scheme 10) [80]. After the U-4CC, the secondary amide linkage was stable and needed to be activated for nucleophile cleavage by *N*-Bocylation (safety-catch principle, cf. Scheme 49). Typical nucleophilic reagents included LiOH and NaOMe. The isocyanide resin **56** was stable for more than six months at low temperature.

**Scheme 11:**
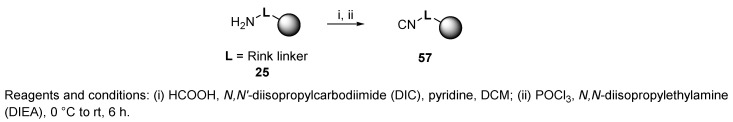
Preparation of convertible Rink-isocyanide resin.

In 2001, Chen et al. first reported the preparation of convertible Rink-isocyanide resin and its usage for the traceless synthesis of 3-acylamido imidazo[1,2-*a*]pyridines [8]. Rink-isocyanide resin **57** (Scheme 11) was prepared via two steps from commercially available Rink resin. The primary amino group of the Rink linker underwent formylation with HCOOH via activation with DIC in the presence of pyridine in DCM, which was followed by dehydration with POCl_3_ in the presence of *N*,*N*-diisopropylethylamine (DIEA) under ice cooling and in an argon atmosphere. The progress of the reaction was monitored by infrared (IR) spectroscopy. The advantage of this kind of resin is its stability (the resin was reportedly stable for over 12 months when stored in a refrigerator) and easy preparation. Moreover, this isocyanide resin is odor-free.

**Scheme 12:**
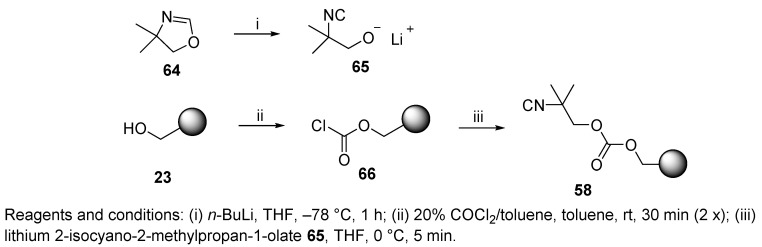
Preparation of the CCI resin.

Another convertible isocyanide used in IMCRs is CCI resin, reported in 2002 by Kennedy and coworkers [81]. Lithium alkoxide **65** (Scheme 12) was prepared from 4,4-dimethyl-4,5-dihydrooxazole **64** and transferred to resin-bound chloroformate **66**, prepared from hydroxymethyl resin **23** and phosgene, to yield the desired isocyanide resin **58**. Treatment of the Ugi product by *t*-BuOK released *N*-acyloxazolidone, which was further reacted with NaOMe in MeOH and yielded the product as an ester (cf. Scheme 48). Polymer-supported carbonate convertible isocyanide **58** could be used to prepare a 4000-membered library.

**Scheme 13:**
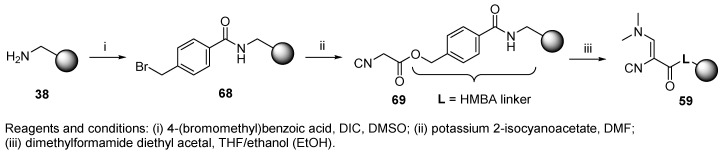
Preparation of 3-*N*,*N*-(dimethylamino)-2-isocyanoacrylate resin.

The last polymer-supported convertible isocyanide discussed in this review is 3-*N*,*N*-(dimethylamino)-2-isocyanoacrylate resin **59** (Scheme 13) [52], which was applied for the synthesis of thiazoles (cf. Scheme 68).

### 3.3. Aldehyde Resin

**Scheme 14:**
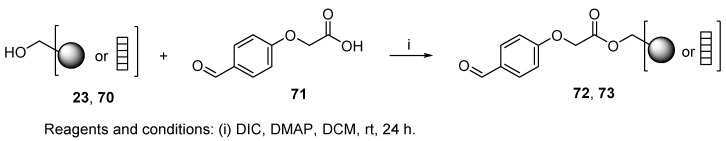
Preparation of aldehyde resin with 2-oxo-1,2-ethylenedioxy linker.

Only one example of aldehyde resin used for U-4CR was reported [82]. The authors attached 2-(4-formylphenoxy)acetic acid **71** (Scheme 14) to hydroxymethyl resin **23** or Lanterns **70** (represented by a “ladder”). The 2-oxo-1,2-ethylenedioxy group served as a linker cleavable either by Yb(OTf)_3_ or TMSCHN_2_ in MeOH or by various amines (cf. Scheme 35).

### 3.4. Carboxylic Acid Resin

**Scheme 15:**
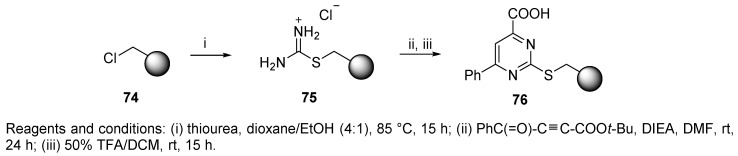
Preparation of immobilized heteroaromatic carboxylic acid.

Carboxylate resin has also not been frequently used, there are only two examples of immobilized carboxylate participating in the U-4CC [40,83]. Thiourea resin **75** (Scheme 15) underwent a cycloaddition reaction with *tert*-butyl-4-oxo-4-phenylbut-2-ynoate, and t-Bu-protected acid was liberated by TFA [40]. The carboxylate resin **76** was further used for the synthesis of various pyrimidine derivatives achieved by multidirectional cleavage (cf. Scheme 36).

**Scheme 16:**
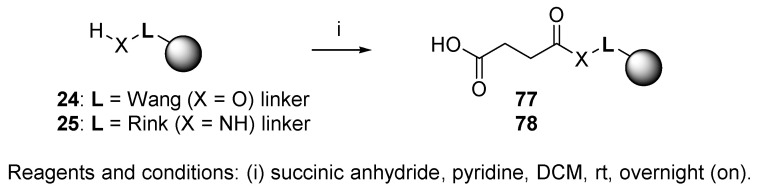
Preparation of immobilized carboxylic acid.

Alternatively, Wang and Rink resins were used to immobilize succinic acid via its anhydride (Scheme 16) [83]. Resin **77** and **78** served as polymer-supported acid inputs in the chemistry of münchnones (cf. Scheme 51).

## 4. Ugi Four-Component Reaction (U-4CR)

In this section, we review the products of classical solid-phase U-4CRs, which are subsequently cleaved from the resin without any other transformation to produce acyclic α-acylamido carboxamides. The formation of two new peptide bonds is the key feature in achieving Ugi products. Therefore, this reaction plays an important role in the synthesis of peptides, including the synthesis of sterically hindered peptides containing α,α-disubstituted glycines (cf. Section 4.1.2) [44]. However, the peptides are not the only compound which can be prepared by U-4CR, as shown in this review.

**Scheme 17:**
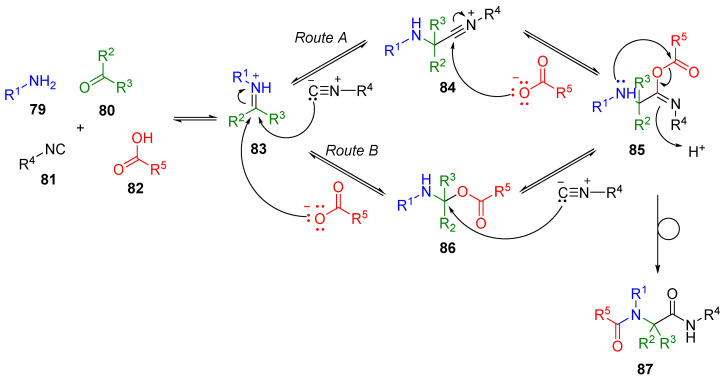
Two plausible mechanisms of the Ugi reaction.

Two possible reaction mechanisms of U-4CR are reported in the literature (e.g., References [84,85]). The originally designed ionic mechanism involved the formation of nitrilium **84** formed in polar protic solvents (typically in MeOH; Route A, Scheme 17). A plausible alternative mechanism considered hemiaminal **86** as the key intermediate (Route B). Resulting imidate **85** subsequently underwent Muum rearrangement, the only irreversible step in this sequence, which shifted the equilibrium to the product side and afforded α-acylamido carboxamide **87**. In the case of phenols being used as an acid input, the reaction sequence is accomplished by the Smiles rearrangement.

### 4.1. α-Acylamido Carboxamides with Three Diversity Positions

#### 4.1.1. Amine Resin

**Scheme 18:**
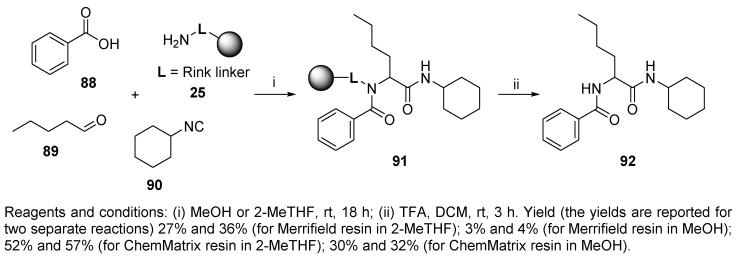
Ugi reaction performed in green solvents.

Recently, green solvents with good swelling properties essential for SPS were applied in the Ugi reaction (Scheme 18) [15]. Merrifield and ChemMatrix resins equipped with a Rink amide linker (loadings 1.0 mmol/g and 0.49 mmol/g, respectively) and two green solvents (MeOH and 2-MeTHF) were selected in this study. As expected, 2-MeTHF was a better solvent for the Merrifield resin-supported Ugi reaction, while the reaction carried out on the ChemMatrix resin was effective in both solvents. This fact is explained by the poor swelling properties of Merrifield resin in MeOH, whereas the polar ChemMatrix resin swells well in both solvents. Reactions with all four combinations of resins and solvents were carried out in duplicate to ensure that the results were reproducible. The Ugi reaction was performed in the solution phase first (the overall yield was 38% in MeOH and 50% in 2-MeTHF). Notably, this extensive study described the swelling properties of nine resins in twenty-five green solvents.

**Scheme 19:**
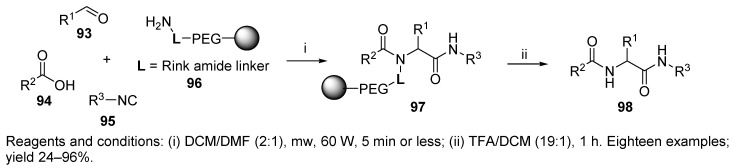
U-4CR of diamide under microwave irradiation.

The second example in this section is the synthesis of diamide **98** (Scheme 19) on TentaGel S RAM resin **96** under microwave irradiation [37]. An eighteen-membered library was prepared by using three aldehydes, three different carboxylic acids, and two isocyanides.

**Scheme 20:**
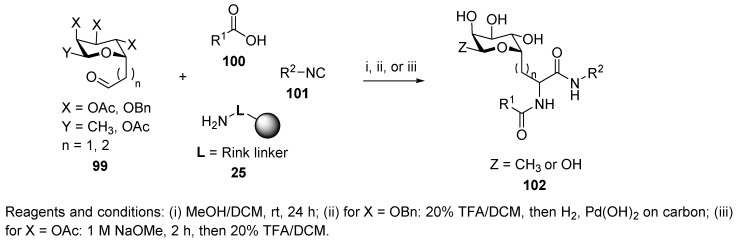
Evaluation of C-glycoside aldehydes in U-4CC.

Armstrong et al. [57] were interested in the synthesis of C-glycoside peptide ligands for cell surface carbohydrate receptors. Prior to synthesizing a library, they carried out a proof-of-concept study focused on a protection strategy (OAc and OBn) of carbohydrates, in which they tested U-4CC with nine C-glycoside aldehydes 99, nine acids, including three C-glycoside acids, nine isocyanides, and Rink resin **25** as an amino-component (Scheme 20). Debenzylation of the cleaved product was carried out by H_2_ with Pd(OH)_2_ on carbon, on resin deacetylation with 1 M NaOMe. Chiral acids had practically no effect on the diastereoselectivity, whereas chiral aldehydes showed the biggest influence due to their proximity to the newly formed stereocenter. The diastereomeric ratio was up to 80:20.

**Scheme 21:**
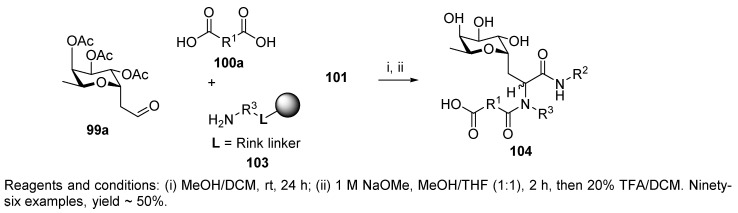
Generation of C-glycoside peptide ligands via U-4CC.

Then, a library of 96 compounds was prepared using Rink resin and five Rink-resin-supported amino acids **103** serving as the amino components, two isocyanides, eight dicarboxylic acids, and one C-glycoside aldehyde (**99a**, Scheme 21). A second library of 192 compounds was prepared using six resin-bound amines, eight diacids, two isocyanides, and one C-fucose as the aldehyde.

**Scheme 22:**
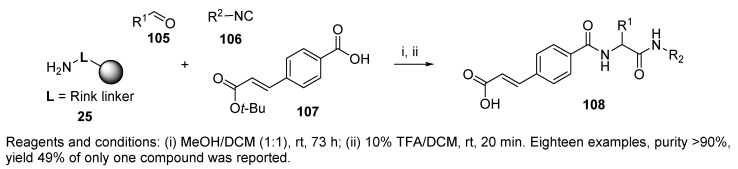
Synthesis of inhibitors of tyrosine phosphatase by U-4CC.

The Ugi reaction also served for the synthesis of peptides used as inhibitors of hematopoietic protein tyrosine phosphatase [69]. The starting compound, carboxylic acid **107** (Scheme 22), was prepared from 4-bromobenzoic acid by reaction with *ter*t-butyl acrylate in the presence of Pd(OAc)_2_ in a yield greater than 90%. The Ugi reaction was followed by cleavage with 10% TFA in DCM. The purity of the liberated products was above 90%. Derivative **108**, where R^1^ = Ph and R^2^ = Bn, was the most active tyrosine phosphatase inhibitor, with ahalf maximal inhibitory concentration (IC_50_) = 3.9 μM.

**Scheme 23:**
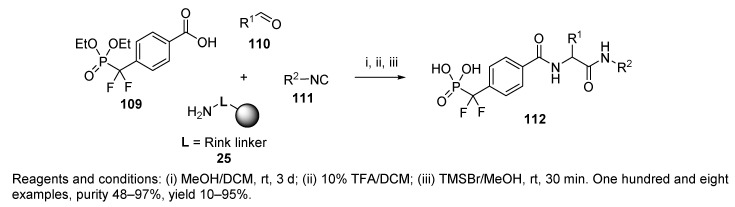
Synthesis of inhibitors of tyrosine phosphatase by U-4CC.

Another study aimed at tyrosine phosphatase inhibitors was dedicated to producing α,α-difluoromethylenephosphonic acids **112** (Scheme 23) [71]. Upon cleavage of the Ugi product from the polymer, the hydrolysis of phosphonate esters with bromotrimethylsilane (TMSBr) was accomplished in the solution phase. Performing the reactions in reverse order was shown to be problematic due to the sensitivity of the Rink resin to acidic conditions. Starting acid **109** was prepared in three steps. Three of the nine tested isocyanides, 1*H*-benzotriazol-1-ylmethyl, 2-morpholinoethyl, and toluene-4-sulfonylmethyl isocyanides, did not result in good yields. The yields of the products fluctuated considerably.

**Scheme 24:**
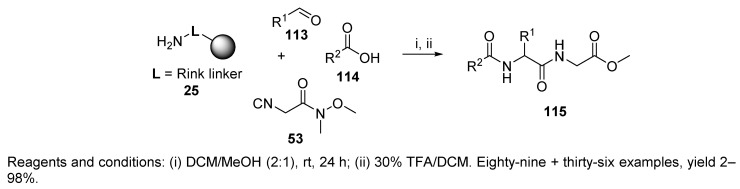
Synthesis of the dipeptide library by the Ugi reaction.

A library of peptides prepared in 96-well microtiter plates was reported by Armstrong and coworkers [35]. Rink resin **25**, eight aldehydes, twelve carboxylic acids, and one isocyanide were combined in the U-4CC (Scheme 24). The yields of product **115** were most sensitive to the nature of the aldehyde component. Aliphatic aldehydes and aldehydes bearing EDG resulted in good yields in comparison to the aldehydes with EWG. Carboxylic acids with phenolic moieties also resulted in poor yields.

The authors further prepared isocyanides, otherwise not commercially available, by quenching α-lithiated isocyanides with alkyl halides. This route opened access to other isocyanides and improved the diversity of the 36-membered library, and the compounds were obtained in 10–55% yield.

**Scheme 25:**
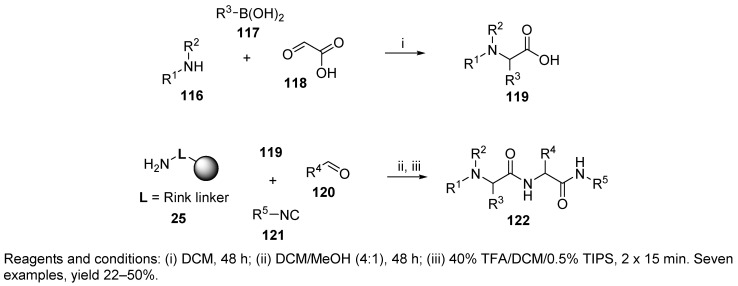
Tandem Petasis/Ugi MCRs leading to highly substituted dipeptides.

A dipeptide was prepared by tandem Petasis/Ugi reactions [58]. Combination of MCRs was first reported in 1993 by Dömling and Ugi [86]. The Petasis reaction, performed in solution, produced α-amino acids **119**, which were subjected to the Ugi reaction with polymer-supported amine **25** (Scheme 25). The Petasis reaction of secondary amine **116**, boronic acid **117**, and glyoxylic acid **118** afforded α-amino acid **119** containing a tertiary amino group. Upon simple removal of DCM, the amino acid could be used for the solid-phase Ugi reaction.

The advantage of this concept is the ability to prepare highly substituted peptides, even on the amine terminus. The authors also applied this strategy with dipeptides and used the Rink-isocyanide resin instead of the Rink resin (cf. Scheme 28).

#### 4.1.2. Isocyanide Resin

**Scheme 26:**
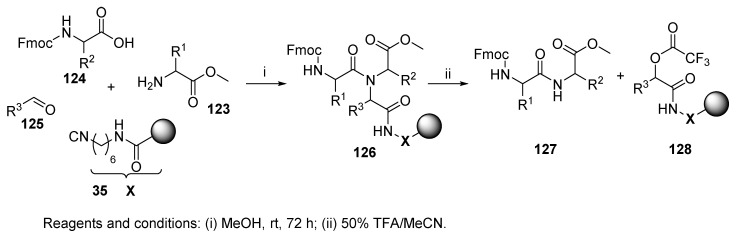
Peptide formation on an isocyanide polymer support by U-4CC.

The first synthesis was carried out using acrylamide-containing resins with various chemical structures, crosslinks, and topographies (cf. Section 2.2) with the aim to synthesize peptides through ligation of a C-terminal fragment with an N-terminal fragment (Scheme 26) [29]. C-protected amino acid/peptide **123** (as an amine component) and *N*-protected amino acid/peptide **124** (as an acid component) were coupled in the presence of an aldehyde and resin-bound isocyanide at room temperature for 72 h. The use of H-Leu-OEt, Boc-Gly-Ala-OH, and 1-methyl-3-formylindole as amine, acid, and aldehyde components afforded tripeptides in approximately 0.5 mmol/g yield. More hindered amino acids, namely, Val-OMe and Fmoc-Leu-OH, yielded dipeptides in amounts of 0.24 mmol/g. Reactions using dipeptides and tripeptides Ala-Val-Leu-OEt with either Cbz-Gly-Phe-OH, Cbz-Gly-Ile-OH, or Boc-Leu-Ala-Gly-OH instead of protected amino acids proceeded less satisfactorily. The yields of the resulting peptides in those cases were approximately 0.05–0.07 mmol/g. The reaction was monitored by IR spectroscopy (by means of comparing the disappearance of the isocyano absorption (~2170 cm^−1^) and the emergence of the ester carbonyl absorption (~1700–1750 cm^−1^) with the intensity of the amide carbonyl moiety absorption (~1650 cm^−1^)). This use of U-4CC for chemical ligation has only recently attracted much attention [16].

**Scheme 27:**
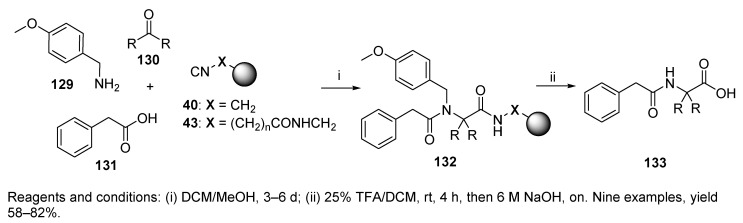
U-4CR in the synthesis of α,α-dialkylglycines.

α,α-Dialkylglycine, a highly hindered C-tetrasubstituted amino acid, was produced by U-4CR [49]. The reaction utilized isocyanide resin either without a linker or equipped with a linker (cf. Scheme 7). The Ugi reaction was carried out with *p*-methoxybenzylamine **129** (Scheme 27), three different ketones **130**, and phenylacetic acid **131** by using isocyanide resins **40** and **43**. The experiment utilizing the resin without any spacer failed to release the target product. In contrast, the Ugi reaction with isocyanide resin equipped with the spacer was successful with all the used ketones (**130**, R = CH_2_CH_3_, (CH_2_)_2_CH_3_, and -(CH_2_)_5_-). The presence of the spacer seemed to be required.

**Scheme 28:**
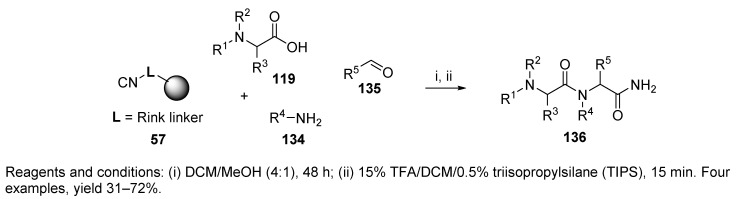
U-4CR involving Petasis product leading to highly substituted dipeptides.

In addition to the synthesis on dipeptides by using Rink resin (cf. Scheme 25), the paper also studied the tandem Petasis/Ugi reaction utilizing universal Rink isocyanide resin **57** (Scheme 28) [58]. The Petasis reactions were carried out in the solution phase and afforded trisubstituted α-amino acid **119**, which was subjected to U-4CC.

### 4.2. α-Acylamido Carboxamides with Four Diversity Positions

#### 4.2.1. Amine Resin

**Scheme 29:**
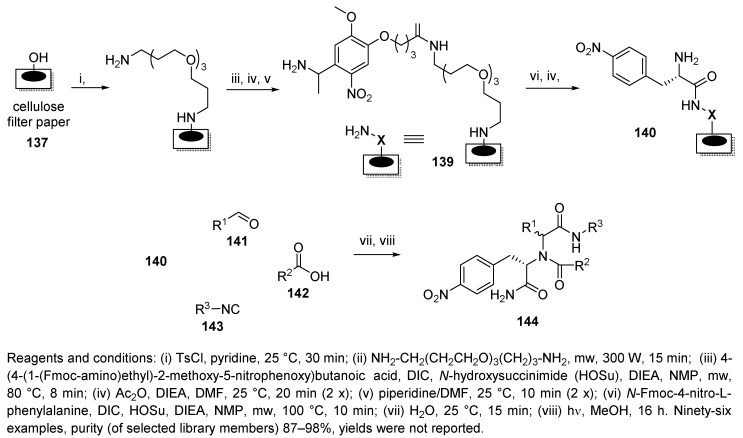
U-4CR using photolabile-linker-modified cellulose.

Whatman filter paper equipped with a photocleavable linker served as a planar cellulose support for the synthesis of small molecule macro-arrays [38]. First, cellulose filter paper was modified with a diamine spacer to enhance its reactivity (**138**, Scheme 29), followed by attachment of the photolabile linker 4-(4-(1-(Fmoc-amino)ethyl)-2-methoxy-5-nitrophenoxy)butanoic acid (**139**). Modified cellulose **139** was then acylated with *N*-Fmoc-4-nitro-L-phenylalanine, which, upon cleavage of the Fmoc group (**140**), could be used for U-4CR. It is important to note that the conversion at room temperature was poor, and neither a longer reaction time, multiple applications of reagents, nor microwave irradiation enhanced the yields. Finally, the rate of the Ugi reaction was successfully accelerated by water [87].

For the construction of a 96-membered macro-array, four aldehydes, six different carboxylic acids, and four isocyanides were chosen. The transformation was most sensitive to the nature of the aldehyde. While aliphatic aldehydes afforded the purest products, aromatic aldehydes were less effective. However, in all cases, the products were obtained in excellent purity. The authors speculated that the photocleavage efficiency was higher with the planar support than with PS beads.

**Scheme 30:**
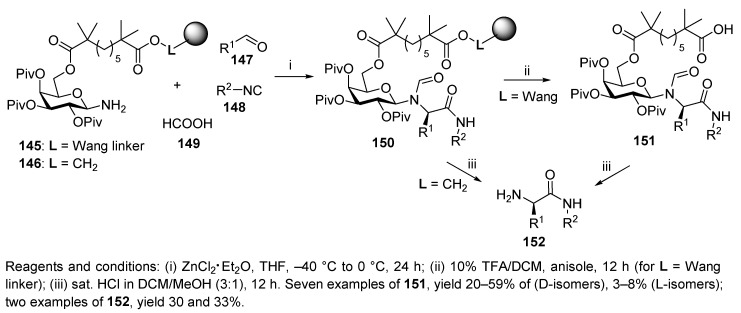
The first diastereoselective Ugi reaction on the solid phase.

Kunz et al. developed the first stereoselective Ugi reaction on the solid phase using carbohydrate as a chiral auxiliary (Scheme 30) [19]. The synthesis sequence started with the laborious six-step preparation of the *O*-pivaloylated polymer-supported galactosylamine **145** and **146**. The Ugi reaction was performed at −40 °C in the presence of zinc chloride etherate. The target products **151** were liberated with 10% TFA in DCM in the presence of anisole and were obtained in good yields and stereoselectivity. The diastereomeric ratios (D:L) of the products **151** ranged from 15:1 to 6:1, and both L- and D-isomers were isolated.

The synthesis was also carried out on Merrifield hydroxymethyl resin. In this case, the resin-bound product was treated with DCM saturated with gaseous HCl in MeOH (3:1) to cleave the *N*-formyl and *N*-glycosidic bonds, releasing the amino acid amide **152**. The same amino acid amide **152** was also obtained from released product **151** upon treatment with HCl.

**Scheme 31:**
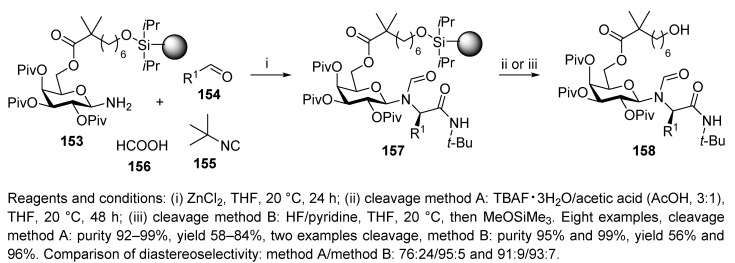
Diastereoselective U-4CR.

Three years later, the same research group applied galactopyranosylamine as a chiral auxiliary in the diastereoselective U-4CR synthesis of *N*-glycosylated *N*-acylated α-amino acid amides (Scheme 31) [20]. U-4CC was carried out with resin-bound galactosylamine **153**, aldehyde, *tert*-butyl isocyanide, and formic acid in the presence of ZnCl_2_. Similar to a previous report [19], the preparation of galactosylamine **153** was laborious. Importantly, the U-4CC proceeded at ambient temperature (in comparison to the previously reported synthesis (Scheme 30)) [19] without any decrease in diastereoselectivity.

The final cleavage was accomplished with TBAF in THF buffered with acetic acid (method A). In the case of intermediates **157** composed of EWG-containing benzaldehyde only, the products were obtained by treatment with a non-basic HF-pyridine complex (method B). The products were obtained in high yield, purity, and diastereoselectivity (diastereomeric ratio (dr) was from 96:4 to 74:26), and the TBAF cleavage caused partial epimerization. Resin-bound galactosylamine **153** was further employed for the synthesis of piperidinones as a core structure in natural products.

**Scheme 32:**
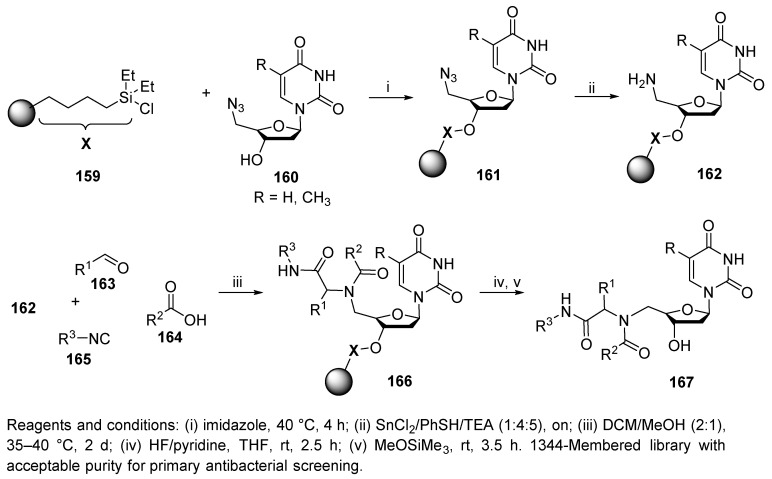
U-4CC in the synthesis of thymidinyl and 2′-deoxyuridinyl nucleoside-peptide library.

A library comprised of 1344 compounds of thymidinyl and 2′-deoxyuridinyl nucleoside-peptides was prepared by the Ugi reaction [72]. The library for antibacterial screening was prepared in fourteen 96-well Flexchem filter plates. The synthesis sequence started with the attachment of 5′-azadinucleoside to commercial PS/DVB-immobilized butyl diethylsilane **159** (Scheme 32). The resulting polymer-supported nucleoside azide **161** was reduced to amine **162**, which was subjected to U-4CR. Product **167** was liberated with HF in pyridine, which avoided any degradation of the nucleoside bond. Methoxytrimethylsilane was used to quench the excess HF.

Notably, an elevated temperature in the Ugi reaction (40 °C) led to higher yields. Additionally, the influences of solvents and molar ratios of the building blocks (BBs) were studied in detail, and the best mixture of solvents was shown to be DCM/MeOH (2:1). The reaction outcomes (reactivity and purity) were most dependent on the nature of the aldehyde (which was consistent with previously reported syntheses, e.g., References [35,36,37]). Aliphatic aldehydes had the highest reactivity, while heterocyclic aldehydes were the least reactive. The Ugi reaction was optimized in a 96-well plate with eight aldehydes, twelve carboxylic acids, one isocyanide, and the resin-bound nucleoside amine.

#### 4.2.2. Isocyanide Resin

**Scheme 33:**
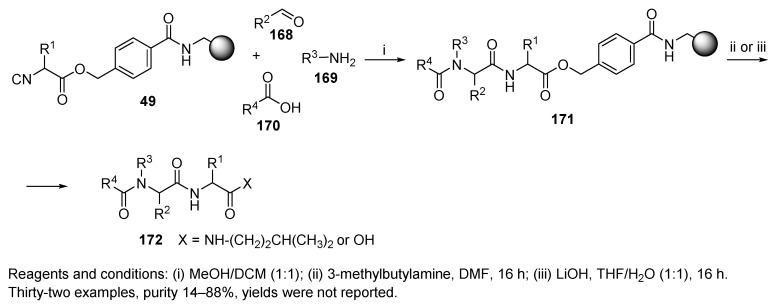
U-4CR involving resin-bound isocyanocarboxylic acids.

A new method for the synthesis of resin-bound isocyanocarboxylic acids **49** (cf. Scheme 8) was described by Henkel and coworkers (Scheme 33) [73]. The Ugi reaction was performed with four resin-bound isocyanides, aldehydes, amines, and acids in a mixture of MeOH and DCM (1:1). After the synthesis, each batch was divided into two parts, and cleaving was performed with two different reagents: 3-methylbutylamine or LiOH. This cleavage procedure liberated amides or carboxylic acids, introducing a fifth element of diversity.

**Scheme 34:**
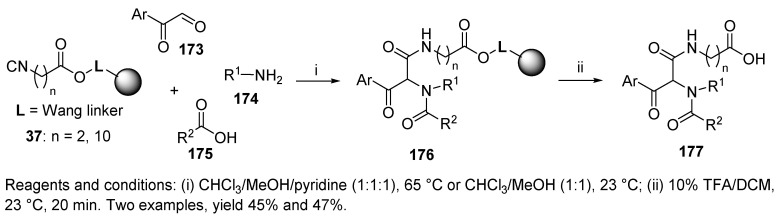
Preparation of α-(*N*-acyl-*N*-alkylamino)-β-keto amides via U-4CC.

Another example of U-4CC with the Wang isocyanide resin **37** (cf. Scheme 6) utilized aryl glyoxal **173**, amine, and carboxylic acid as starting BBs (Scheme 34) [50]. The main aim of this work was to achieve the cyclization of polymer-supported Ugi product **176** to imidazoles **337** (cf. Scheme 57).

#### 4.2.3. Aldehyde Resin

**Scheme 35:**
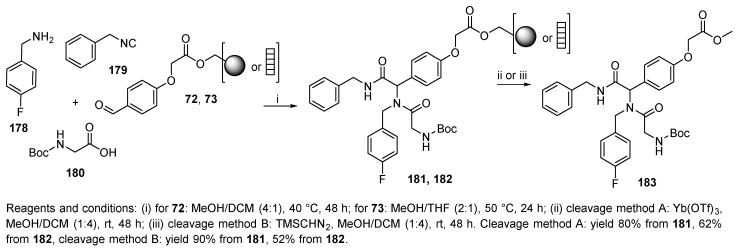
Ugi reaction in the synthesis of drug-like small molecules.

The following work was aimed at the discovery of drug-like small molecules, potent agonists for growth hormone secretagogue (Scheme 35) [82]. The Ugi reaction was performed in the solution phase as well as on three different supports, namely, soluble methoxy poly(ethylene glycol), and two solid supports (hydroxymethyl resin **23** and SynPhase^®^ Lanterns **70**, cf. Scheme 14). A crosslinked polymer with a 2-oxo-1,2-ethylenedioxy linker (**72** and **73**) was used for the Ugi reaction with N-Boc-glycine, 4-fluorobenzylamine, and benzylisocyanide. Polymer-supported α-(acylamino)carboxylic acid esters **181** and **182** were subsequently exposed to two different cleavage agents: Yb(OTf)_3_ (method A) or TMSCHN_2_ (method B). Although the cleavage conditions were mild, trimethylsilyl diazomethane is highly toxic.

The solid-supported reactions required heating (40–50 °C). The Ugi reaction on hydroxymethyl resin-bound aldehyde was completed within 24–48 h, while the reaction with Lantern-immobilized aldehyde was terminated after 24 h and prolonging the reaction time did not lead to better yields. The yield of the products was improved by the addition of a catalytic amount of perchloric acid. Both solid supports were quantitatively recovered after the cleavage of the product. This approach was then applied to the synthesis of a potent agonist for growth hormone secretagogue.

#### 4.2.4. Carboxylic Acid Resin

**Scheme 36:**
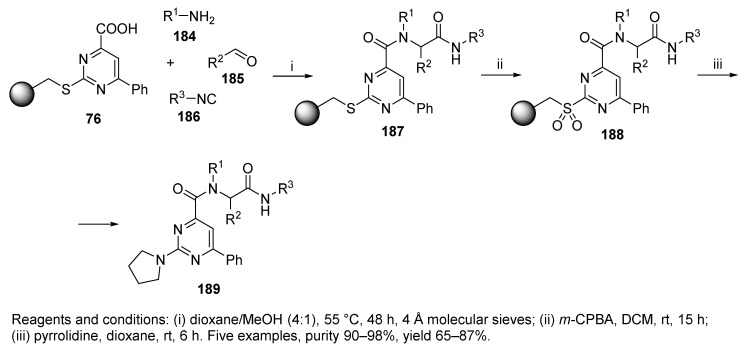
U-4CR utilizing polymer-supported carboxylic acid.

The next application of U-4CR utilizing polymer-supported carboxylic acid **76**, which was prepared from the Merrifield resin via three steps (cf. Section 3.4), yielded resin-bound pyrimidines, which were liberated by oxidation with *m*-CPBA followed by nucleophilic displacement with pyrrolidine (product **189**, Scheme 36) or with other N-nucleophiles to expand the diversity [40]. This method of cleavage is known as multifunctional (multidirectional) cleavage [88] and utilizes a safety-catch linker [89]. A description of this methodology is included in a recently published review [90].

## 5. U-4CR Followed by Cyclative Cleavage

**Figure 3:**
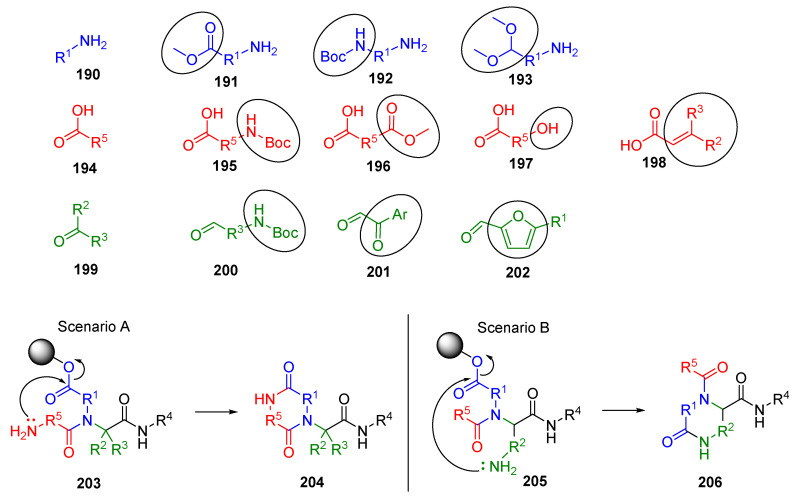
Examples of bifunctional BBs and ring closures.

Cyclative cleavage is considered the most effective method for releasing the products from the insoluble support. Typically, an internal nucleophile (nitrogen or oxygen) attacks an ester-type bond of an acyclic U-4CC product with the resin, which results in the release of cyclic products. The reaction conditions are usually mild, and only the cyclic product is released; thus, the crude purity is generally high. This methodology opens access to six- and seven-membered cycles. The following illustration shows the bifunctional BBs (the functional group involved in the post-Ugi transformation is circled) and an example of two scenarios of cyclization (Figure 3).

### 5.1. Diketopiperazines

**Scheme 37:**
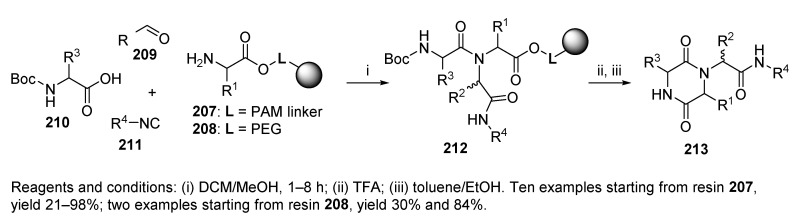
Synthesis of diketopiperazines.

Diketopiperazines were synthesized via the Ugi reaction either on classical solid-phase support (resin beads) [6,45,46], or on cellulose filter paper [62]. Two different approaches leading to diketopiperazines were reported by Szardenings et al. [46]. The first one involved a five-step synthetic sequence (not included in this review), while the second one utilized U-4CC followed by cyclative cleavage (product **213**, Scheme 37) and afforded products in higher yields. Two solid supports were tested, namely, 4-(hydroxymethyl)phenylacetamidomethyl (PAM) **207** and TentaGel S-OH **208** resins. The latter polymer afforded the products in higher yields, while the reaction on PAM resin required DCE as a cosolvent. The yields were mostly dependent on the aldehyde. While the aliphatic aldehydes reacted within minutes, aromatic aldehydes and ketones (not shown in the scheme) required a reaction time of several hours. It is important to note that the Ugi reaction tolerated hindered amino acids, such as valine, in contrast to the classical approach consisting of a five-step synthetic sequence. The authors also synthesized diketomorpholines (cf. Scheme 45).

**Scheme 38:**
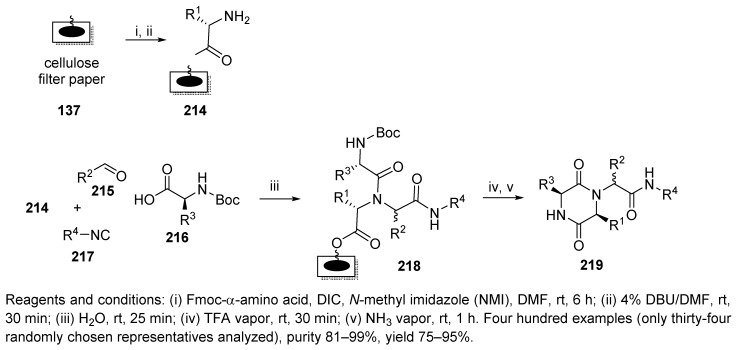
SPS of diketopiperazines via the UDC strategy.

The Ugi/deBoc/cyclization (UDC) strategy, first reported by Hulme and coworkers [91], was applied for the synthesis of a 400-membered library of diketopiperazines intended as luminescence inhibitors of the bacterial symbiont Vibrio fischeri [62]. Cellulose membrane (Whatman chromatography paper) **137** was used as a solid support, and neither a spacer nor a linker was required to attach the first BB, Fmoc-α-amino acid (Scheme 38). Deprotection of the amino group (**214**) was followed by U-4CR with five different Boc-α-amino acids, four aldehydes, and four isocyanides. Linear Ugi-product **218** triggered acid-mediated Boc cleavage, which was followed by spontaneous cyclative cleavage to produce target diketopiperazines **219**. Gaseous ammonia was used to deprotonate the amino group.

**Scheme 39:**
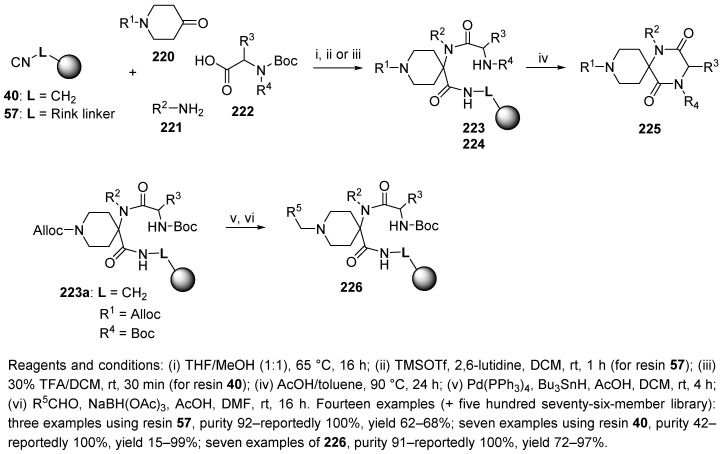
Synthesis of spirodiketopiperazines as potent, selective CCR5 antagonists.

A 576-member library of spirodiketopiperazines as potent, selective CCR5 antagonists, was readily prepared through U-4CR [45]. Two different isocyanide resins (**40** and **57**, Scheme 39) were based on aminomethyl and Rink amide resins, respectively. In the Ugi reaction, cyclative cleavage of monocyclic precursors **223** and **224** was induced at elevated temperature in the presence of acetic acid. To expand the diversity at the R^1^ position, an Alloc-protected piperidinone was incorporated (resin **223a**). The Alloc-protecting group was removed, the secondary amine was reductively alkylated with an aldehyde (resin **226**), and the product was released upon Boc removal. For hit identification, the Ugi reaction was performed with eight piperidinones, nine amines, and eight amino acids to prepare a 576-member library. The synthesis was also studied in the solution phase.

**Scheme 40:**
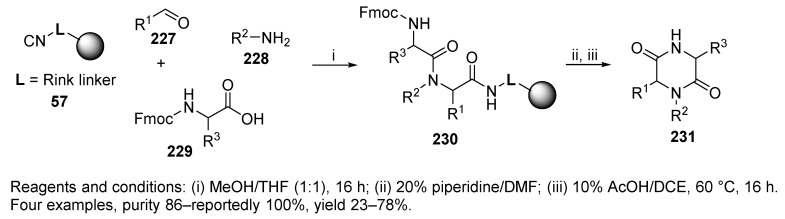
Diketopiperazines prepared on universal Rink-isocyanide resin.

Universal Rink-isocyanide resin **57** (Scheme 40) was utilized for U-4CR, leading to diketopiperazines **231** [6]. U-4CR yielded linear resin-bound α-acylamino carboxamides **230**, which was deprotected. Cyclative cleavage in 10% AcOH in DCE at elevated temperature led to the desired diketopiperazines **231** with excellent purity. The formation of benzodiazepines **258** and 5-substituted 1*H*-tetrazoles **433** was also investigated (cf. Schemes 44 and 73, respectively).

### 5.2. Ketopiperazines

**Scheme 41:**
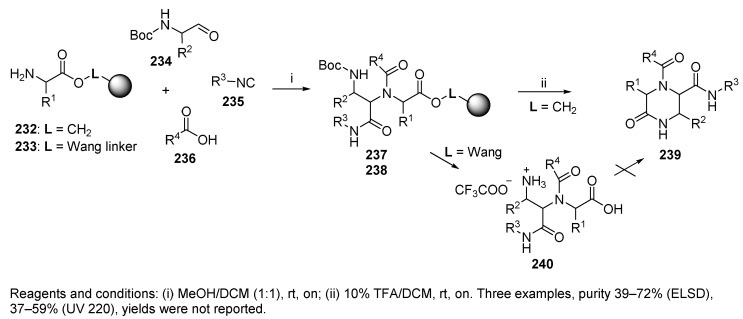
Synthesis of ketopiperazines via the UDC strategy.

Hulme et al. applied the UDC strategy to synthesize ketopiperazines **239** (Scheme 41) and 1,4-benzodiazepine-2,5-diones **252** (cf. Scheme 43) as potential fibrinogen receptor antagonists and anticonvulsant and hypocholesteremic agents [4]. The synthesis of ketopiperazines **239** was performed on an acid-stable hydroxymethyl resin because the ester bond was not cleaved by TFA, and the release and formation of target ketopiperazines **239** occurred via a cyclative cleavage mechanism. The application of the Wang resin led to cleavage of acyclic derivative **240**, which did not cyclize in solution. Interestingly, the synthesis of benzodiazepines **252** showed the opposite effect (cf. Section 5.4). The purity was judged by two methods: evaporative light scattering detection (ELSD) and UV detection at 220 nm.

### 5.3. Bicyclic Diketopiperazines

**Scheme 42:**
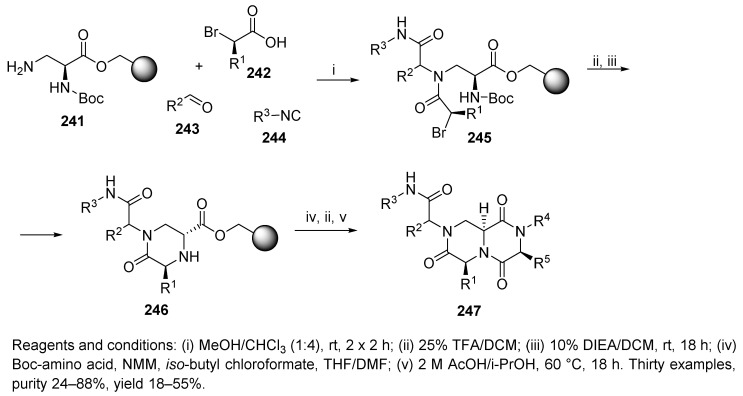
Ugi reaction as the key step in the synthesis of β-turn peptidomimetics.

On the basis of a previously published strategy yielding peptide β-turn mimetics [59], Golebiowski et al. synthesized a library of bicyclic diketopiperazines as putative β-turn peptidomimetics [36]. The key step was the Ugi 4-CC reaction of α-*N*-Boc-diaminopropionic acid ester, which was prepared via the Mitsunobu reaction and attached to a Merrifield-type hydroxymethyl resin (**241**) with α-bromoacid **242**, aldehyde, and isocyanide (Scheme 42). The poor diastereoselectivity of the Ugi 4-CC reaction resulted in a mixture of diastereomers (1:1 to 1:2) at the R^2^ stereocenter; however, this approach did allow for the control of all remaining chiral centers. Cyclative cleavage occurred at elevated temperatures in the presence of acetic acid.

### 5.4. Benzodiazepines

**Scheme 43:**
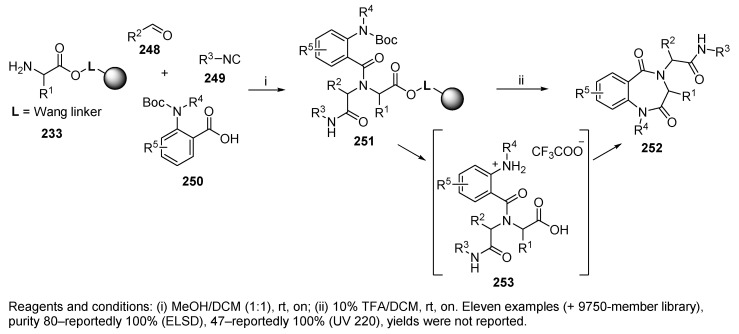
Synthesis of 1,4-benzodiazepine-2,5-diones via the UDC strategy.

In addition to ketopiperazines **239** (cf. Scheme 41), a synthetic route to produce benzodiazepines **252** (Scheme 43) was developed by Hulme and coworkers [4]. 4-CR of the amino acid attached to the Wang resin via carboxylate **233**, aldehyde, isocyanide, and *N*-substituted-*N*-Boc-anthranilic acid **250** resulted in the polymer-supported Ugi product **251**, which, upon treatment with 10% TFA in DCM and evaporation at room temperature in a SAVANT^TM^ evaporator (otherwise isocyanide-derived amide bond was hydrolyzed to carboxylic acid), resulted in target benzodiazepines **252** in good to excellent yield. In contrast to the case of ketopiperazines (cf. Scheme 41), acyclic intermediate **253** was not detected, probably due to rapid cyclization to the target compounds. A cyclocleavage mechanism was also considered.

Notably, the synthesis of benzodiazepines was also performed in the solution phase; however, the solid-phase approach was preferable due to the tendency of methyl esters to form ketopiperazines in solution. The synthesis was applied to produce a 9750-member library by using eight amino acids, eight *N*-Boc-anthranilic acids, ten isocyanides, and fifteen aldehydes.

**Scheme 44:**
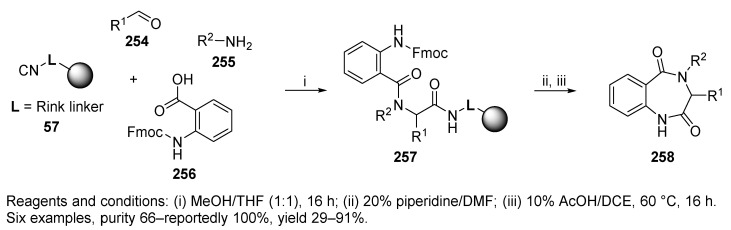
Benzodiazepines prepared on universal Rink-isocyanide resin.

In addition to the previously mentioned diketopiperazines **231** (cf. Scheme 40), universal Rink-isocyanide resin **57** was utilized for U-4CR with Fmoc-anthranilic acid **256**, leading to the acyclic Ugi products **257** (Scheme 44) [6]. Acid-mediated cyclative cleavage resulted in the formation of target benzodiazepines **258** in variable yields.

### 5.5. Diketomorpholines

**Scheme 45:**
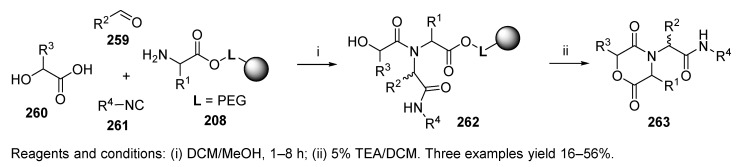
Synthesis of diketomorpholines.

Diketomorpholine **263** (Scheme 45) [46] was produced by the Ugi reaction with α-hydroxycarboxylic acid **260** in place of N-Boc-protected amino acids, which afforded the previously mentioned diketopiperazines (cf. Scheme 37). The solid-phase Ugi reaction was performed with TentaGel S-OH resin because it afforded products in higher purity in comparison with the Wang or PAM resins. Cyclative cleavage of **262** induced by an O-nucleophile was accomplished with 5% TEA in DCM, and 95% TFA in H_2_O [92] resulted in numerous side products.

## 6. Post-Ugi Transformations of Convertible Isocyanides

**Scheme 46:**
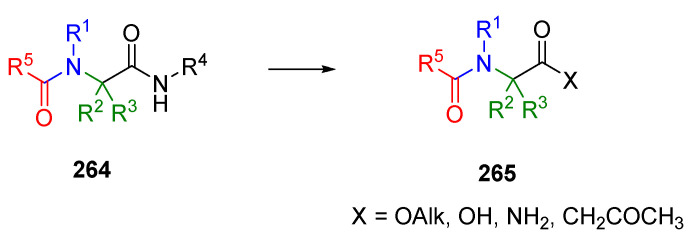
Transformations of convertible isocyanide.

The unavailability of diverse isocyanides from commercial sources and the need to synthesize unpleasant odorous (although not particularly toxic) starting synthons triggered the development of universal isocyanides. The term universal refers to the ability to modify the secondary amide incorporated by isocyanide after completing U-4CC. This approach aimed to make the amide bond susceptible to cleavage under mild conditions while not affecting the other amide. The R^4^ amide (Scheme 46) was transformed into esters [1,61,80,81,83], carboxylic acids [80,83,93], and ketones [2]. The post-Ugi transformation strategy incorporates a fourth diversity element without the need to prepare a diverse set of isocyanides. The transformed product can be used for further modification (e.g., heterocycle formation). The convertible isocyanide can be either attached to the resin or used as one of the components in the solution.

### 6.1. Polymer-Supported Convertible Isocyanides

**Scheme 47:**
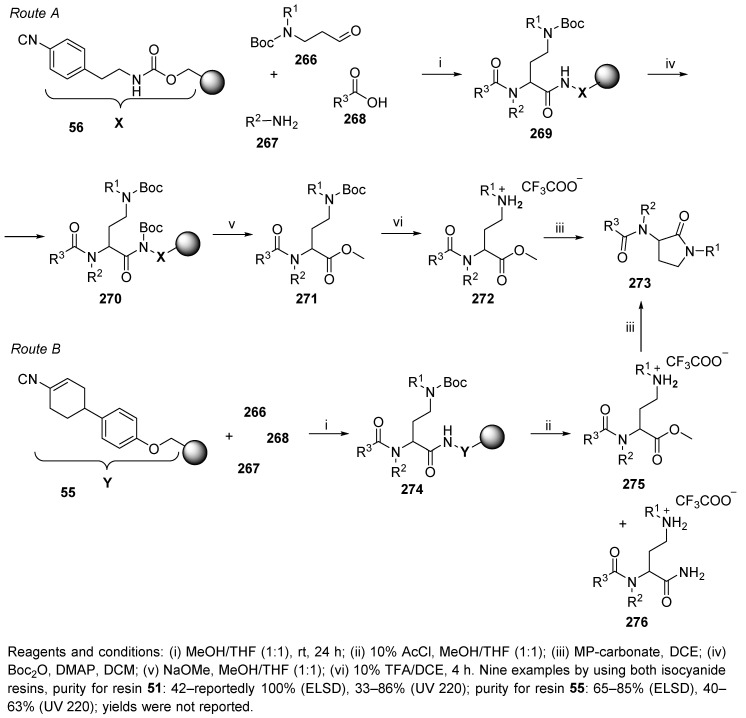
Synthesis of γ-lactams via the UDC strategy.

Hulme et al. applied convertible isocyanides to synthesize γ-lactam analogs [1]. The synthesis utilized a UDC strategy and was performed in solution as well as on the solid phase. U-4CR was carried out on versatile safety-catch linker **56** [80] (Route A, Scheme 47) and on resin-bound cyclohexenyl isocyanide **55** (Route B), with N-Boc-β-amino-aldehydes **266** (easily prepared from the carboxylic acid via the Weinreb amide formation and subsequent reduction with LiAlH_4_) [94], amines, and carboxylic acids.

Route A involves the reaction of polymer-supported Ugi product **269** with Boc_2_O (resin **270**) to activate the isocyanide-derived carboxamide for a nucleophilic attack by NaOMe. Liberated methyl esters **271** were treated with 10% TFA in DCE (linear intermediate **272**) and yielded the target lactams **273**. The cyclization was promoted by solvent evaporation at 65 °C in a SAVANT^TM^ evaporator. Alternatively, the Ugi product bound to the cyclohexenyl resin (intermediate **274**) was converted to methyl ester **275** and subsequently cyclized to product **273** (Route B). The partial cyclization was completed by a proton scavenger. This route suffered from the formation of a side product, carboxamide **276**. The purities were analyzed by ELSD and UV absorption analysis at 220 nm.

Both isocyanide resins were stable at –20 °C and odorless. In contrast, the stability of the *N*-Boc-β-amino-aldehydes was limited (it is necessary to use freshly prepared batches). In this paper, the synthesis of bicyclic γ-lactams was carried out (cf. Scheme 64).

**Scheme 48:**
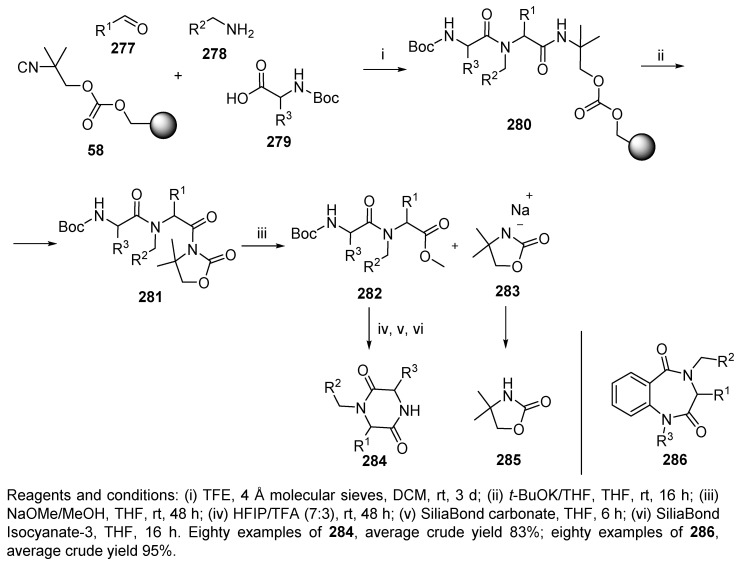
Synthesis of diketopiperazines and benzodiazepines by using universal isocyanide resin.

In 2002, Kennedy et al. discovered a novel CCI resin **58** (cf. Scheme 12) and demonstrated its applicability to the synthesis of 2,5-diketopiperazines **284** and 1,4-benzodiazepine-2,5-diones **286** (Scheme 48) [81]. The synthesis sequence comprised U-4CC followed by a two-step, one-pot procedure (including base-activation of the resin-bound Ugi product **280** resulting in cleavage via formation of a *N*-acyloxazolidone **281**, which was then converted to methyl ester **282**). Final post-cleavage cyclization in a mixture of HFIP (1,1,1,3,3,3-hexafluoropropan-2-ol) and TFA led to the desired 2,5-diketopiperazines **284**. The crude product was treated with silica-based scavengers to remove impurities. Similarly, 1,4-benzodiazepine-2,5-diones **286** were prepared (in the Ugi reaction, *N*-R^3^-anthranilic acids were used).

**Scheme 49:**
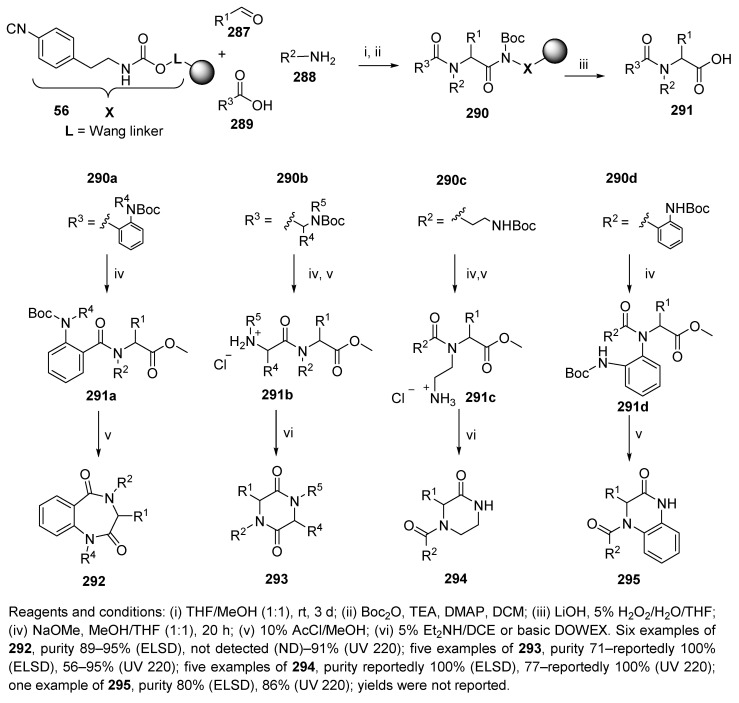
Synthesis of nitrogen-containing heterocycles on Wang resin-bound isocyanide.

The use of safety-catch linker **56** (cf. Scheme 10) allows for cleavage of the product upon Boc activation with a nucleophile (methoxide or hydroxide) and was reported by Hulme and coworkers [80]. The UDC strategy was applied to the preparation of carboxylic acids **291**, 1,4-benzodiazepine-2,5-diones **292**, diketopiperazines **293**, ketopiperazines **294**, and dihydroquinoxalinones **295** (Scheme 49). The Ugi reaction of the Wang resin-bound isocyanide **56**, which was prepared via four steps (cf. Scheme 10), aldehyde, amine, and carboxylic acid yielded α-acylamido carboxamides, which needed to be activated by Boc_2_O prior to cleavage (resin **290**). Four different internal N-nucleophiles were tested, namely, *N*-Boc anthranilic acid, *N*-Boc α-amino acids, and two *N*-Boc diamines. Cleavage of the Boc group with AcCl in MeOH and evaporation at 65 °C in a SAVANT^TM^ evaporator afforded the target bicycles **292** and **295**. Partial cyclization to **293** and **294** was facilitated by a base. The purities were analyzed by ELSD and UV absorption analysis at 220 nm.

### 6.2. Convertible Isocyanides in Solution

**Scheme 50:**
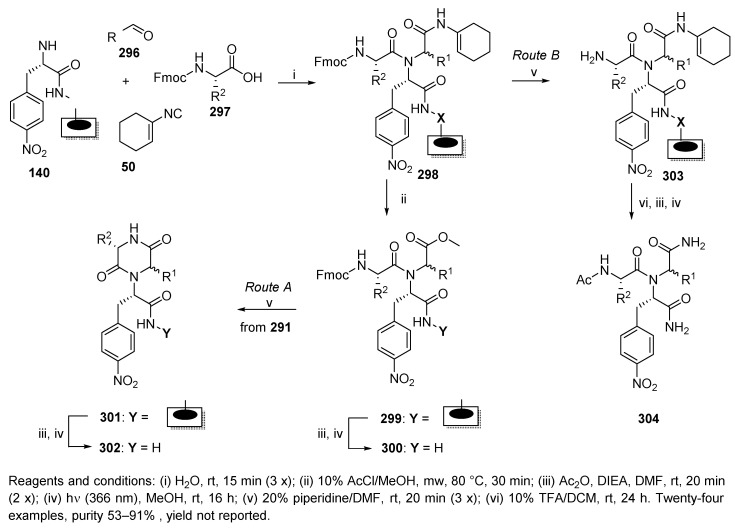
Synthesis of diketopiperazines via Ugi 4-CR and subsequent cyclization.

The planar cellulose support, previously described for the synthesis of peptides (cf. Scheme **29**) [38], was also utilized for the preparation of diketopiperazine macro-arrays [61]. This synthesis was inspired by reports on syntheses carried out in solution [77], and on the solid phase (cf. Scheme 37) [46]. Solid-supported Ugi product **298** (Scheme 50) was converted into methyl ester **299** [78], which, upon cleavage of the Fmoc group, underwent spontaneous cyclization to immobilized diketopiperazines **301** (Route A). Subsequent treatment with Ac_2_O in the presence of DIEA and photocleavage afforded the target diketopiperazines **302**. When intermediate **298** was directly subjected (after cleavage of the Fmoc group) to cyclization with 10% TFA in DCM, as reported by Hulme [77], the anticipated product was not obtained (Route B). Only primary amide hydrolysis product **304** and other by-products were formed. The Ugi reaction was performed with three aldehydes and eight amino acids.

**Scheme 51:**
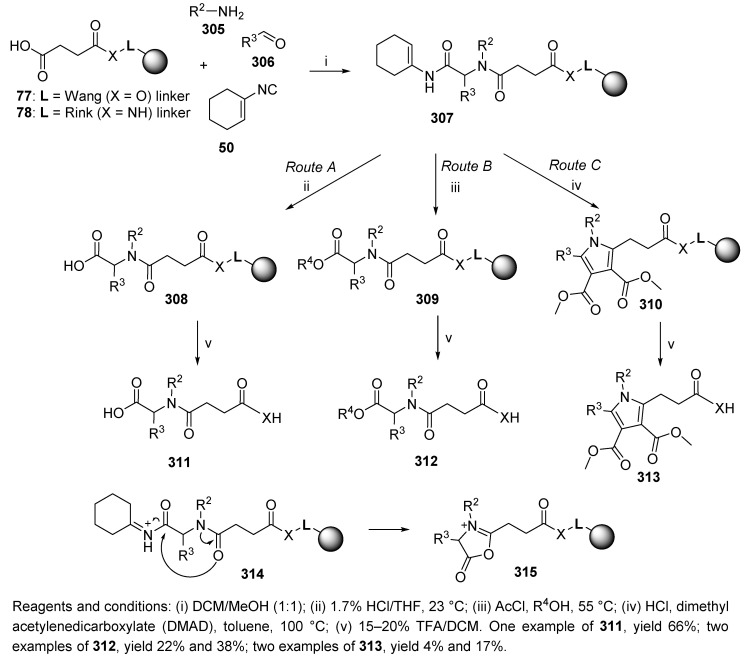
Conversion of the Ugi product to carboxylic acids, esters, and pyrroles via münchnones.

The U-4CC of resin-bound carboxylic acids **77** and **78** (cf. Scheme 16), amine, aldehyde, and 1-isocyanocyclohexene, resulted in cyclohexenamides **307** (Scheme 51) [83]. They were subsequently transformed into carboxylic acids **308**, esters **309**, or pyrroles **310**, which, upon acidic cleavage, resulted in the desired products **311**–**313**. The synthetic strategy was based on 1-isocyanocyclohexene as a convertible isocyanide which afforded unrelated products.

It is important to note that the intermediate formed in each route was münchnone **315**, which was formed by cycloelimination of protonated cyclohexenamide **314**. The münchnone then underwent a nucleophilic attack by water or alcohol, leading to carboxylic acid **311** or ester **312** (Route A and Route B), or proton cleavage, causing the formation of a 1,3-dipole, which is capable of cycloaddition with acetylene (product **313**, Route C).

**Scheme 52:**
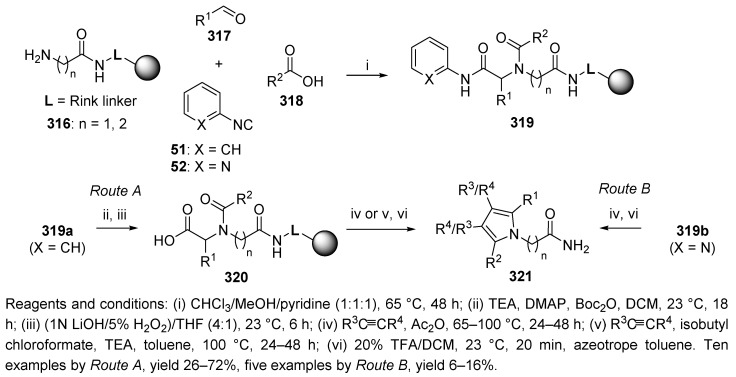
Conversion of the Ugi product to pyrroles via münchnones.

The transformation of münchnones with respect to the formation of carboxylic acids and pyrroles was also described by another research group [93]. Mjalli et al. carried out U-4CC of polymer-supported amine **316**, aldehyde, carboxylic acid, and isocyanides **51** and **52**, which afforded *N*-acyl-*N*-alkyl-α-amino amides **319** (Scheme 52). The reaction was carried out in a mixture of CHCl_3_/MeOH/pyridine (1:1:1), in which the pyridine buffered the reaction mixture and stabilized the isocyanide.

Following hydrolysis of Ugi product **319a** (X = CH) to the corresponding acids, **320** proceeded in two steps (Route A). The target pyrroles **321** were achieved by a 1,3-dipolar cycloaddition of the alkynes with the resin-bound münchnones. Alternatively, polymer-supported amide **319b** (X = N, Route B) was cyclized directly to münchnone, which was trapped in situ with different alkynes to yield **321**. Route B produced the pyrroles in lower yields; on the other hand, it proceeded via a single step.

**Scheme 53:**
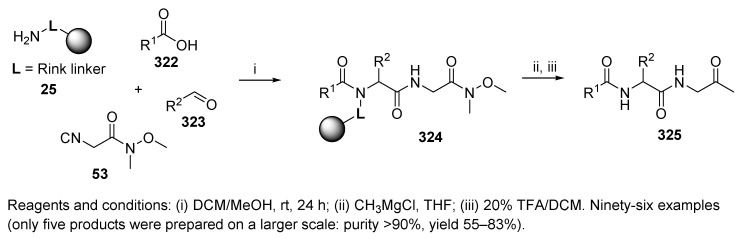
U-4CC with a Weinreb-type amide followed by the Grignard reaction.

Armstrong et al. reported the Ugi four-component synthesis of diamide backbone **324** followed by derivatization through displacement of a Weinreb amide [95] with a Grignard reagent (Scheme 53) [2]. Acidic cleavage led to the target α-oxodipeptides **325**. A 96-membered library was prepared by using twelve carboxylic acids and eight aldehydes. The effect of these components on the yields of the target products was studied. Aliphatic aldehydes or aldehydes bearing EDG afforded the products in good yields in comparison to those derived from EWG-containing aldehydes. While carboxylic acids with phenolic moieties resulted in low yields, the others led to products in good yields. The larger scale did not significantly influence the yields. The Weinreb amide was prepared from glycine via six steps in solution.

## 7. Other Post-Ugi Transformations

In this section, we include transformations utilizing bifunctional components for the synthesis of Ugi products and exploiting the appended functional group for ring formation. These chemistries include cyclic iminium formation [34], ring-closing metathesis [56], Diels-Alder reaction [3,96], and imidazole synthesis from β-keto amides [50].

### 7.1. Spontaneous Transformations

**Scheme 54:**
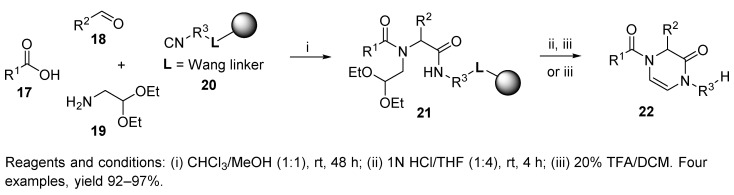
Synthesis of ketopiperazines via N-acyliminium ion formation.

Spontaneous transformations proceed simultaneously with the cleavage of the acyclic Ugi products from the resin. The first example is the formation of ketopiperazines by tandem U-4CC followed by *N*-acyliminium ion cyclization [34]. Wang resin-bound isocyanide (**20**, R^3^ = *p*-CH_2_-C_6_H_4_-O, Scheme 54) entered the Ugi reaction with a carboxylic acid, aldehyde, and aminoaldehyde diethyl acetal. Acidic treatment of polymer-supported Ugi product **21** cleaved the acetal, led to the formation of *N*-acyliminium ion, followed by the loss of proton upon neutralization, and resulted in the target unsaturated heterocyclic product **22**. Note, HCl treatment prior to TFA cleavage was not necessary. The synthesis was reported in the solution phase as well.

**Scheme 55:**
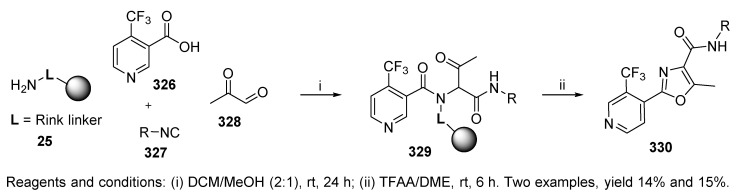
U-4CC followed by Robinson-Gabriel synthesis of oxazoles.

2,4,5-Trisubstituted oxazoles, which are known pesticides, were synthesized via α-acylamino ketones **329** subjected to the Robinson-Gabriel intramolecular cyclization by using TFAA in DME, where TFAA served as both a cleavage and cyclodehydration agent (Scheme 55) [70]. In addition to affording α-acylamino ketones by U-4CC, five more synthetic strategies were investigated. Despite low yields (39% and 40% crude, 14%, and 15% after passage through a silica gel plug), the Ugi reaction was beneficial due to the single step-formation of α-acylamino ketones. Only two examples were given.

**Scheme 56:**
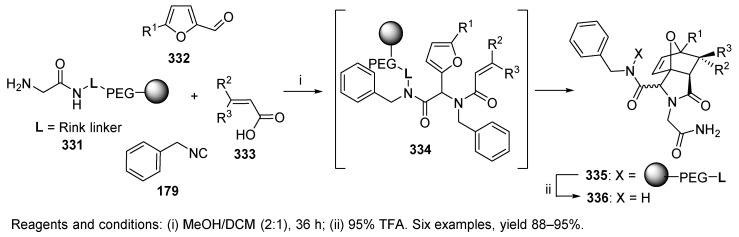
Tandem 4-CC/intramolecular Diels-Alder reaction to rigid tricyclic lactams.

The Diels-Alder reaction is another example of a post-Ugi transformation. Resin-bound amine **331**, which was prepared via two steps from ArgoGel Rink resin, furalaldehyde **332**, benzylisocyanide **179**, and activated dienophile acids **333** (such as maleic and fumaric acid derivatives), led to the formation of triene **334**, which immediately underwent an acid-mediated intramolecular Diels-Alder reaction to afford highly functionalized rigid tricyclic lactams **335** (Scheme 56) [96].

The influence of electronic and steric effects was investigated. While condensation of unsubstituted furaldehyde (**332**, R^1^ = H) led to products in high yields and good diastereoselectivities (the ratio of the two isomers ranged from 91:9 to 88:12), 5-methylfuraldehyde (R^1^ = CH_3_) afforded products with moderate diastereoselectivity (the ratio of the two isomers ranged from 88:12 to 77:23). The synthesis was initially carried out in the solution phase.

### 7.2. Mediated Transformations

**Scheme 57:**
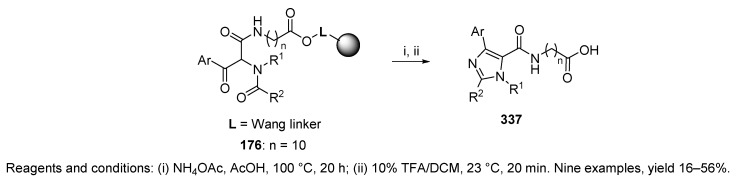
Synthesis of tetrasubstituted imidazoles.

This section discusses syntheses involving additional step(s) following the Ugi product formation prior to the final cleavage from the resin. The first example in this section utilized a Wang resin-bound isocyanide (cf. Scheme 6) in the Ugi reaction leading to α-(*N*-acyl-*N*-alkylamino)-β-keto amides **177** (cf. Scheme 34) [50]. Immobilized Ugi products **176** underwent cyclization to imidazoles in NH_4_OAc and AcOH at 100 °C, and simultaneous release from the resin with 10% TFA in DCM led to the target tetrasubstituted imidazoles **337** (Scheme 57). The effects of the BBs on the reaction outcomes were studied. While aniline resulted in low yield (16%), isobutylamine, benzylamine, and even 4-methoxyaniline resulted in moderate yields (35–56%). Aryl glyoxals were tolerated (both unsubstituted and 4-methoxy-substituted) as well as carboxylic acids (aliphatic and aromatic). Finally, the length of the isocyanide linker had no effect on the yield of the imidazoles. Notably, some products were transformed to their methyl esters prior to purification. Direct exposure of Ugi product **176** to 10% TFA in DCM led to the corresponding amides **177** (cf. Scheme 34).

**Scheme 58:**
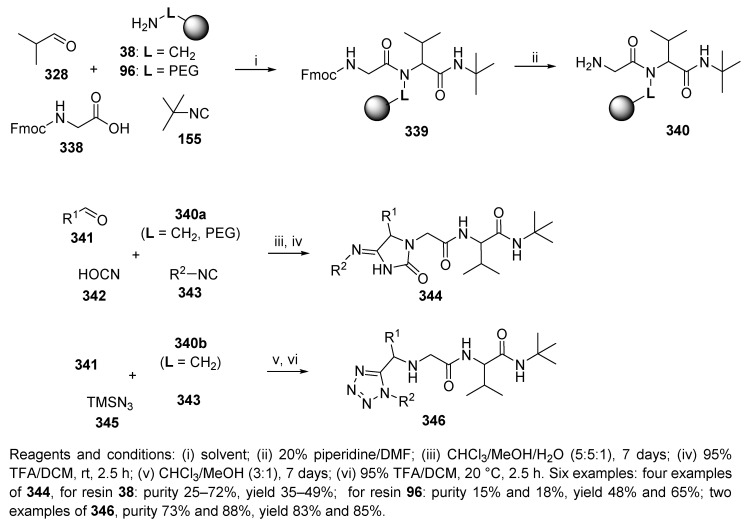
Repetitive Ugi reaction.

Ugi and his coworker prepared hydantoinimide **344** and tetrazole **346** derivatives by repetitive Ugi reactions using aminomethyl PS resin **38** (Scheme 58) [60], and the hydantoinimides were also synthesized on TentaGel resin **96**. A sequence of two different Ugi 4-CRs was employed. The first Ugi reaction was performed with α-amino acid (Gly), and the subsequent Ugi reaction was carried out with cyanic acid and trimethylsilyl azide as alternative acid inputs (more details about the alternative acid reagents can be found in Section 9.2).

Hydantoinimides **344** were obtained in low purities (20–40%) when aromatic aldehyde (benzaldehyde) was used. In contrast, the purity of tetrazole derivative **346** was 88% when benzaldehyde was used. The synthesis sequence was also performed in solution.

**Scheme 59:**
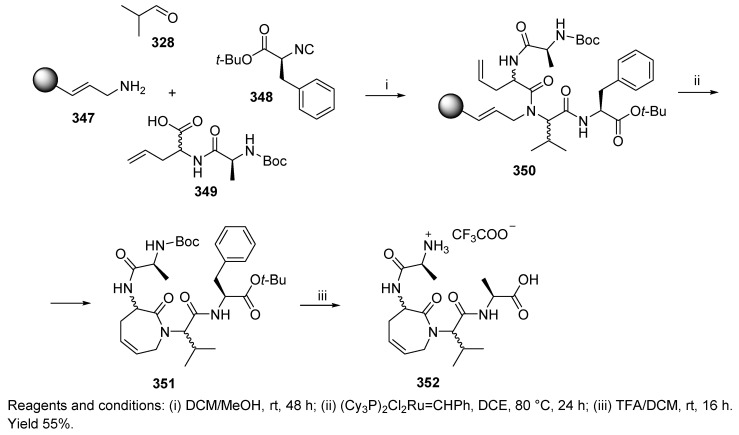
Tandem Ugi 4-CR followed by ring-closing metathesis leading to β-turn mimetics.

Synthesis of β-turn mimetics of the Freidinger lactam type was also achieved with U-4CC (Scheme 59) [56]. Acyclic tetrapeptide **350** was then subjected to ring-closing metathesis, including carbon–carbon bond formation and concomitant cyclative cleavage, and yielded lactam ring-containing product **351**. Acid labile protecting groups were cleaved upon TFA treatment (**352**).

**Scheme 60:**
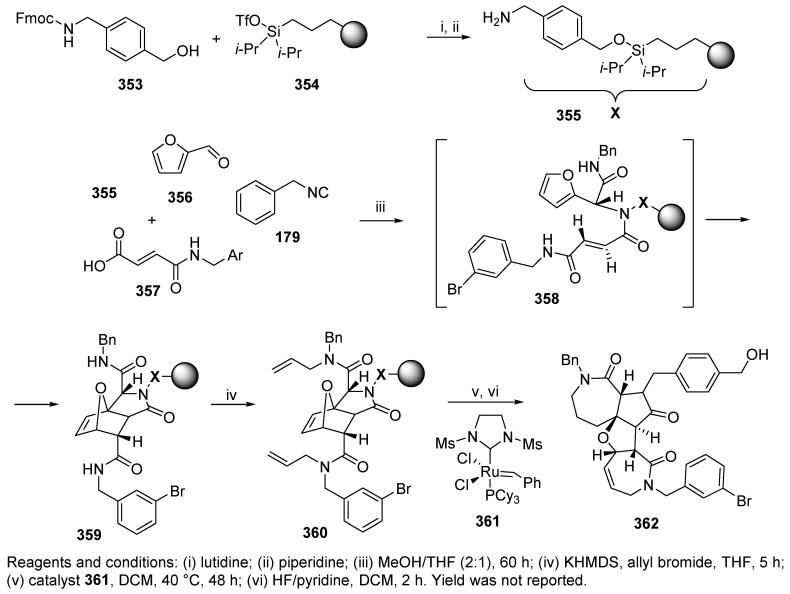
Formation of a 7-5-5-7 polycyclic ring including U-4CC.

An illustrative example of the application of the Ugi reaction to generate structural complexity in synthetic products was demonstrated by Schreiber et al. [3]. Complex structures were generated via a minimal number of steps: Ugi 4-CR, intramolecular Diels-Alder reaction, and ring-opening-closing olefin metathesis, and all these steps were performed on PS modified with carbon-and-silicon-only extender **354** (Scheme 60). The Ugi reaction with immobilized amine **355**, furfural **356**, benzyl isocyanide **179**, and fumaric acid derivative **357** was carried out in a mixture of MeOH/THF at a ratio of 2:1. Resin-bound Ugi product **358** immediately underwent an intramolecular Diels-Alder reaction, and intermediate **359** was subsequently treated with potassium hexamethyldisilazide (KHMDS) and allyl bromide in THF (**360**). Finally, the metathesis (20 mol% of catalyst **361**, DCM, 40 °C) was followed by the release of product **362** with HF/pyridine in DCM. The synthesis sequence was initially performed in the solution phase, with a yield of 69%.

## 8. Ugi Four-Center Three-Component Reaction (U-4C-3CR)

**Figure 4:**
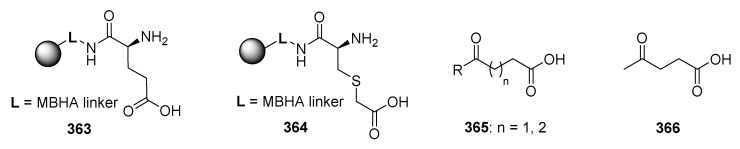
Bifunctional BBs used in U-4C-3CR.

This strategy is desirable from the cyclization point of view: the product of this reaction is a heterocycle. The cyclization is achieved by a single input compound containing two functional groups required for the Ugi reaction. To date, only two types of bifunctional components have been reported, linking an acid with an amine and an acid with a carbonyl group. The bifunctional components were either resin-bound or in solution (Figure 4).

### 8.1. Resin-Bound Bifunctional Reagents

**Scheme 61:**
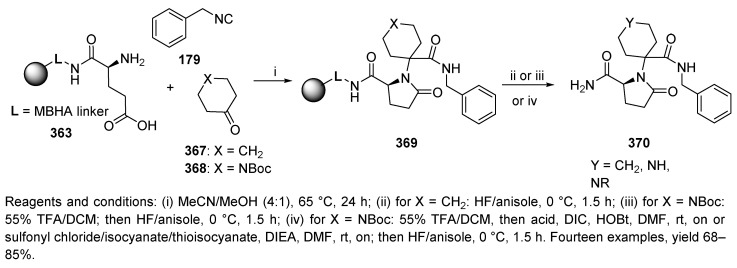
Synthesis of N-substituted pyrrolidinone by U-4C-3CR.

Liu and Nefzi described the synthesis of N-substituted pyrrolidinone-tethered *N*-substituted piperidines via U-4C-3CR [47]. The synthesis sequence started with MBHA-resin-supported glutamic acid **363**, which reacted with two ketones and four isocyanides (Scheme 61). However, the Ugi reaction was not successful with resin-bound aspartic acid as the bifunctional reagent.

**Scheme 62:**
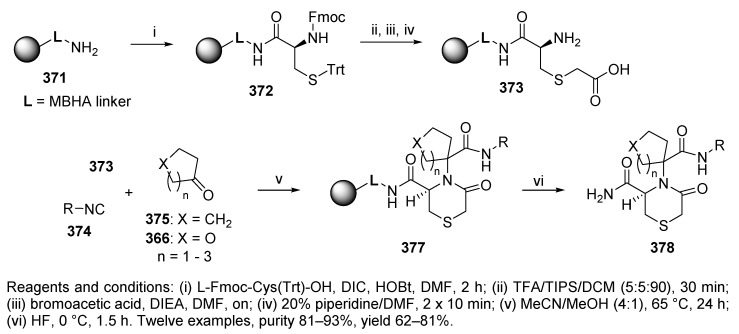
Synthesis of *N*-substituted thiomorpholinones by U-4C-3CR.

Liu and Nefzi also carried out the synthesis of thiomorpholines via Ugi 4C-3CR [48]. MBHA resin-bound bifunctional BB **373** was prepared via four steps and reacted with four symmetrical ketones and three different isocyanides at 65 °C overnight (Scheme 62). The target enantiopure thiomorpholines **377** were cleaved with HF.

### 8.2. Bifunctional Reagents in Solutions

**Scheme 63:**
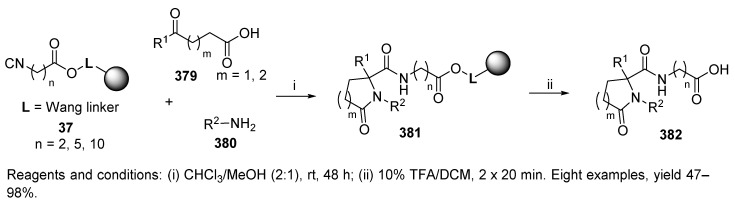
U-4C-3CR leading to lactams.

Keto acids with different chain lengths **379** served as bifunctional reagents in the synthesis of lactams **382** (Scheme 63) [54]. Their reaction with resin-bound isocyanide **37 [50]** and amines yielded ɣ- and δ-lactams **381**, which were released with 10% TFA in DCM. The reaction was also performed in the solution phase.

**Scheme 64:**
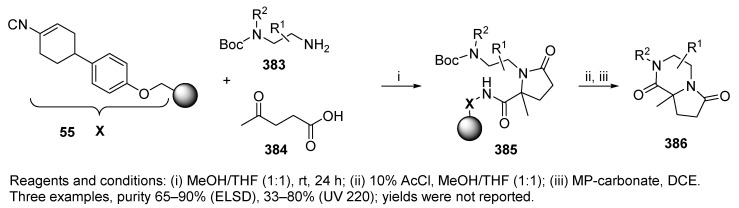
Synthesis of bicyclic γ-lactams.

Hulme et al. [1] reported on U-4C-3CR using cyclohexenyl isocyanide resin **55** (Scheme 64), *N*-Boc diamines **383 [97]**, and a bifunctional reagent, levulinic acid **384**. The Ugi reaction was followed by post-condensation modification affording fused lactam-ketopiperazines **386**.

## 9. Non-Traditional Ugi Reaction

In this section, we include IMCRs with one of the inputs, amine or acid, replaced by a different reactant. The amine was replaced with sulfonamide, and the acid was replaced with thiocarboxylic acid, alcohol, or inorganic acid, including HOCN, HSCN, or HN_3_. In addition, we incorporate modified Ugi reactions that did not fit into any of the above-used categories.

### 9.1. Alternative Amine Input

#### Sulfonamide

**Scheme 65:**
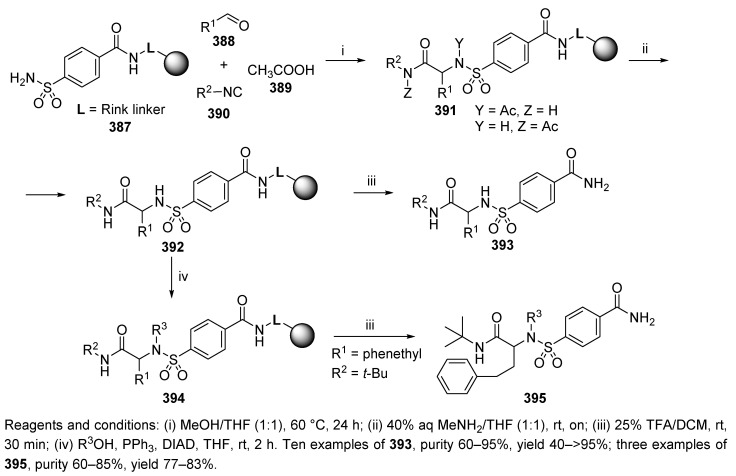
Ugi-type 4-CC involving polymer-supported arylsulfonamide.

The two following examples were reported by the Lou laboratory [43]. α-Amino acid sulfonamides were synthesized by Ugi-type 4-CC utilizing polymer-supported sulfonamide as the amine input (**387**, Scheme 65). The synthesis was carried out in the solution phase and was not successful. Even after 3 days, the majority of the sulfonamide was unreacted, and the only a trace amount of the product was observed. The SPS improved the reaction rate, and the best results were obtained at elevated temperature (60 °C) in 24 h. In addition to the expected product **391**, a small amount of deacetylated product was also observed. Cleavage of the acetyl group was then accomplished in 40% aqueous (aq) MeNH_2_ and THF at a ratio of 1:1 (resin **392**). The target products **393** were obtained with 25% TFA in DCM. The diversity of the products was expanded by *N*-alkylation under Mitsunobu conditions (product **395**).

**Scheme 66:**
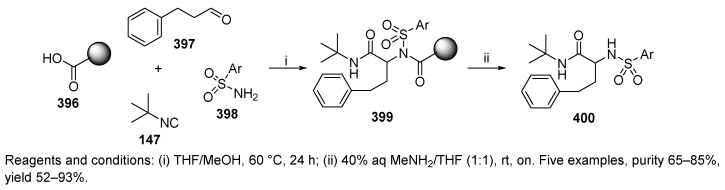
Ugi-type 4-CC involving arylsulfonamide in solution.

Analogous sulfonamides, structurally complementary to the products obtained with the first approach (Scheme 65), were achieved by using carboxy PS **396** (Scheme 66) [43]. Ugi 4-CC was followed by traceless cleavage to result in the target products **400**.

### 9.2. Alternative Acid Input

#### 9.2.1. Thiocarboxylic Acid

**Scheme 67:**
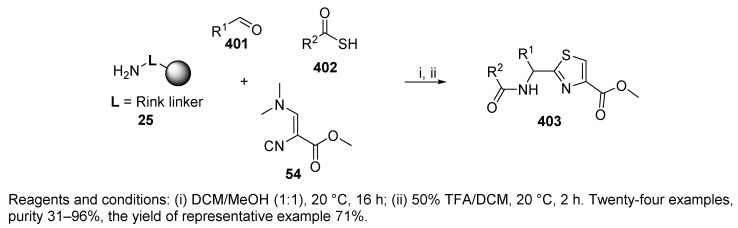
Synthesis of thiazoles on Rink amide resin.

In addition to the previously reported solution-phase U-4CR leading to thiazoles [98,99], Dömling et al. [51] conducted another study on the solid-phase Ugi reaction of 3-*N,N*-(dimethylamino)-2-isocyanoacrylate **54** (Scheme 67) and thiocarboxylic acid **402**, which yielded 4-carboxy-2-acylaminomethylthiazoles **403**. Reagent **54**, described for the first time in 1979 [100], represents rich functional isocyanide, bearing an isocyano functionality, a Michael acceptor, and dimethylamino leaving group.

The synthesis was compatible with both aliphatic and aromatic thiocarboxylic acids as well as with 16 tested aliphatic and (hetero)aromatic aldehydes. A potential drawback of the usage of thiocarboxylic acids is their limited commercial availability [98]. On the other hand, there are many methods for their preparation (e.g., Reference [101]).

**Scheme 68:**
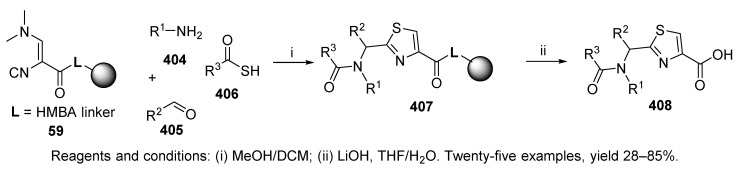
Synthesis of thiazoles via resin-bound 3-*N*,*N*-(dimethylamino)-2-isocyanoacrylate.

In a follow-up synthesis, Dömling et al. applied 3-*N*,*N*-(dimethylamino)-2-isocyanoacrylate attached to resin **59** (for its preparation, cf. Scheme 13) to synthesize thiazoles **408** (Scheme 68) [52]. Thiobenzoic acid afforded slightly higher yields than thioacetic acid.

#### 9.2.2. (Thio)cyanic Acids and Hydrogen Azide

Replacing carboxylic acids with inorganic acids, such as HOCN, HSCN, or HN_3_, leads to alternative nitrogenous heterocycles [53,54].

**Scheme 69:**
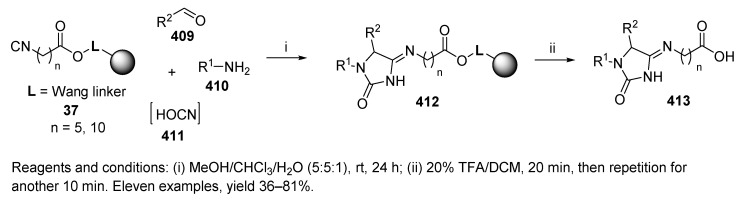
Preparation of hydantoin 4-imides by U-4CC.

U-4CC of Wang resin-bound isocyanide **37** [50], aldehyde, amine, and in situ generated HOCN **411** (from pyridine hydrochloride and KOCN), led to hydantoin 4-imides **412** (Scheme 69) [53]. Several amines and aldehydes were tested. While the reaction tolerated different types of amines, it was highly dependent on the nature of the aldehyde. Aliphatic aldehydes (branched and unbranched) worked for this reaction; in contrast, aromatic aldehydes did not afford the products. One year later, the chemistry was extended and also described in the solution phase [54].

**Scheme 70:**
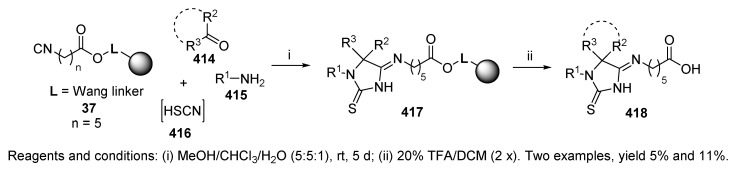
Synthesis of 2-thiohydantoin 4-imides by U-4CR.

This synthetic strategy was adopted for the preparation of 2-thiohydantoin 4-imides **418** (Scheme 70). Cyanic acid was replaced by thiocyanic acid **416** (prepared in situ), and aldehyde was replaced by ketone (aldehyde was reportedly unreactive). Disappointingly, the Ugi reaction with these components afforded very low yields of **418** (5% and 11%).

**Scheme 71:**
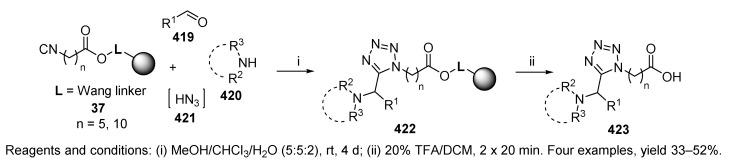
Synthesis of 5-(1′-aminoalkyl)tetrazoles.

Mjalli et al. [54] investigated the reaction of polymer-supported isocyanide resin **37** (Scheme 71) [50], aldehyde, primary or secondary amine, and hydrogen azide **421** generated in situ. The Ugi reaction resulted in the formation of resin-bound tetrazoles **422**, which were cleaved with 20% TFA in DCM. Both primary and secondary amines were tolerated as well as *p*-bromoaniline. In contrast to the Ugi reaction producing hydantoin 4-imides **413** and **418** (Scheme 69 and Scheme 70), aromatic aldehydes were compatible, although the yields were mediocre.

#### 9.2.3. Alcohol

**Scheme 72:**
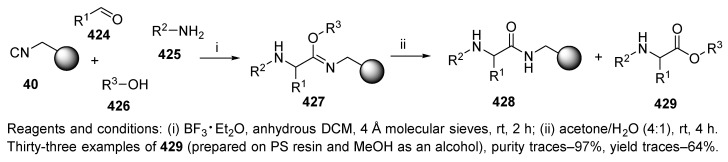
4CR in the synthesis of *N*-substituted α-amino acid esters.

Henkel and coworkers employed 4-CR to synthesize *N*-substituted α-amino acid esters [39]. The outcome of the synthesis was based on the aqueous cleavage of imino-ethers, which depended on the reaction conditions. In addition to α-amino acid amides **428** (Scheme 72), which were generated in most cases, α-amino acid esters **429** were also formed.

The Ugi reaction of isocyanide resin **40** [74] with aldehydes, amines, and alcohols, led to polymer-supported imino-ethers **427**, which are key intermediates. When isocyanide tethered to aminomethyl PS resin was used, product **429** was not formed unless the reaction was catalyzed. Therefore, the synthesis was performed in the presence of a Lewis acid catalyst, boron trifluoride diethyl etherate. Cleavage in an aqueous acetone solution liberated α-amino acid esters **429**, while the products of hydrolysis, α-amino acid amides **428**, remained on the resin.

Both yields and purities were higher when the aldehyde and amine were pre-condensed to the imine. Additionally, aromatic amines led to better results with respect to purity and yield than aliphatic amines. In contrast, the type of alcohol had no influence on the reaction outcome (methanol, isopropanol, and phenylpentanol resulted in comparable outcomes). The reaction was also performed by using TentaGel resin, which improved the results compared to hydrophobic PS resin. Seventeen of the thirty-three successfully prepared α-amino acid methyl esters were obtained in ≥90% crude purity. However, the yields were mediocre.

#### 9.2.4. Azide

**Scheme 73:**
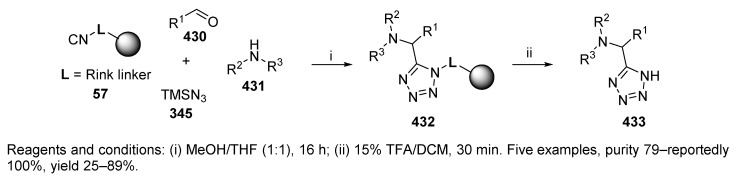
5-Substituted 1*H*-tetrazoles prepared on universal Rink-isocyanide resin.

Four-component reactions involving aldehydes, amines, isocyanides, and trimethylsilyl azide, leading to the formation of tetrazoles, are referred to as Ugi-azide-4CRs. Tetrazoles are considered bioisosteres of carboxylic acids and were shown to preserve or improve biological activities. The first example is the previously mentioned repetitive Ugi reaction (cf. Scheme 58), and the second example is the solid-phase Ugi-azide-4CR generating 5-substituted tetrazoles by using convertible Rink-isocyanide resin [6]. Resin-bound tetrazoles **432** underwent acidic cleavage to produce the target 5-substituted tetrazoles **433** (Scheme 73). Notably, secondary amine was used instead of traditional primary amine. This is an example of traceless synthesis [90].

**Scheme 74:**
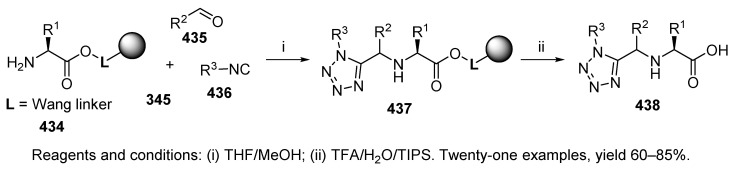
Synthesis of tetrazole-peptidomimetics by Ugi-azide 4CR.

Rivera et al. used Ugi-azide-4-CR to synthesize a library of tetrazole-peptidomimetics, which are potent inhibitors of the *Escherichia coli* M1-aminopeptidase [5]. α-Amino acid attached to Wang resin **434** (Scheme 74) was reacted with aldehyde, isocyanide, and trimethylsilyl azide **345**. Four different amino acids, five aldehydes, and three isocyanides were used to generate a set of 1,5-disubstituted tetrazoles **438**. The products were obtained as a mixture of two diastereomers (dr was from 1:1 to 4.7:1).

### 9.3. U-4CR Using Preformed Schiff Base

**Scheme 75:**
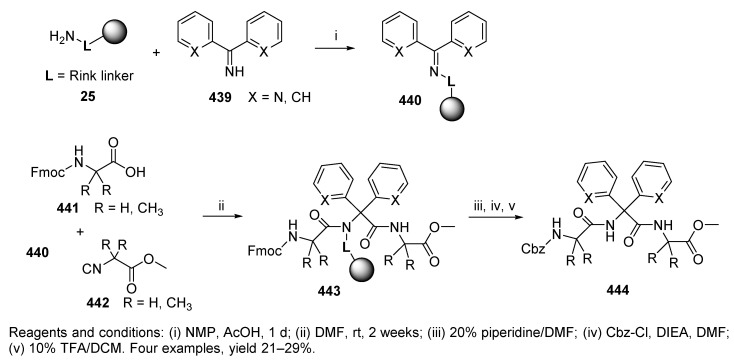
Synthesis of tripeptides by modified Ugi reaction.

In addition to the classical U-4CR, a modified approach was investigated for the preparation of tripeptides with α,α-disubstituted glycine with two pyridine rings **444** (Scheme 75) [44]. The difficulty of reacting sterically hindered amine and diaryl ketone was overcome by preforming Schiff base iminium ions to participate in the Ugi-type reaction (with isocyanide and carboxylic acid).

Polymer-supported Schiff base **440** was reacted with Fmoc-amino acids (Fmoc-Gly-OH or Fmoc-4-aminoisobutyric acid) and isocyanide **442**. The Fmoc group of the resulting resin-bound Ugi product **443** was replaced with a Cbz protecting group, and the product was cleaved from the resin to yield tripeptide **444**. The yields of bis-pyridyl tripeptides obtained from the solid-phase approach were higher than those obtained from solution-phase synthesis. The bis-phenyl derivatives showed the opposite trend. The effect of the solvent used in the Ugi reaction was investigated and the best results were obtained with DCM and NMP or 2,2,2-trifluoroethanol as a cosolvent. The drawback of this synthesis is its very long reaction time. It is worth noting that α,α-disubstituted glycine with two pyridine rings served as a very effective peptide backbone constraint [102].

## 10. Other IMCRs

### 10.1. Passerini Three-Component Reaction (P-3CR)

The Passerini reaction is a three-component reaction between aldehydes, carboxylic acids, and isocyanides, which leads to α-acyloxy carboxamides. Passerini and Ugi reactions are concurrent reactions, and the Passerini product is often observed as a side-product of U-4CC. In contrast to the Ugi reaction, which is typically performed in polar protic solvents, the Passerini reaction is favored in nonpolar solvents. The two reported examples from the same laboratory [10,11] aimed at the preparation of β-acyalamino-α-hydroxyamides and β-acyalamino-α-oxoamides containing peptides, known as protease inhibitors.

**Scheme 76:**
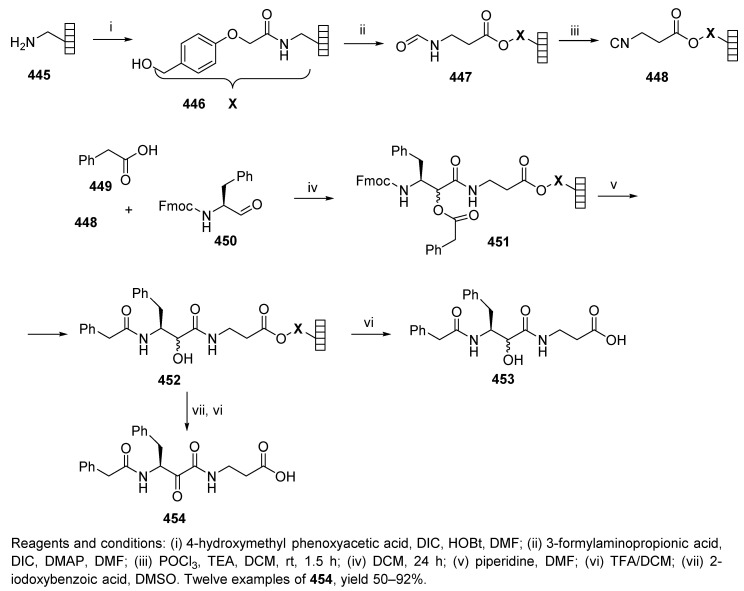
Synthesis of oligopeptides through P-3CR.

The sequence of the reported transformations involved the Passerini reaction, amine deprotection, and acyl migration (PADAM). P-3CR was performed on aminomethyl Lantern^®^ **445** (Scheme 76) acylated with 2-(4-(hydroxymethyl)phenoxy)acetic acid to form Wang-type Lanterns **446**. Solid-supported isocyanides **448** were prepared via three steps. The resin-bound linker was acylated with 3-formylaminopropionic acid followed by dehydration to form Lantern-bound isocyanide **448**. Treatment of Passerini product **451** with piperidine induced Fmoc cleavage and simultaneous acyl migration. Intermediate **453** was then cleaved, and β-acylamino-α-hydroxyamide **453** was achieved with dr 6:4. Oxidation of **452** followed by treatment with TFA led to the target ketopeptide **454 [11]**. Twelve products were prepared from the isocyanides derived from three amino acids (β-Ala, Ala, Leu), two aldehydes derived from Fmoc-Phe and Fmoc-Ala, and two acids (phenylacetic and hippuric acids). The same synthetic strategy was applied to the synthesis of peptidomimetics on aminomethyl PS modified with a photocleavable linker [10].

### 10.2. Groebke-Blackburn-Bienaymé Three-Component Reaction (GBB-3CR)

**Scheme 77:**
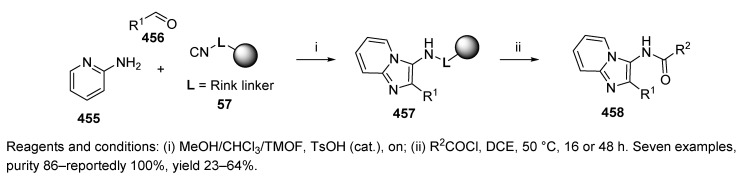
Synthesis of 3-substituted imidazo[1,2-*a*]pyridines by GBB-3CR.

GBB-3CR is a reaction between aldehyde, isocyanide, and amine-containing NH_2_-C=N moieties in their cyclic structure (2-aminoazine or amidine). This reaction was efficient in the preparation of imidazo[1,2-*a*]-annulated pyridines, pyrazines, and pyrimidines as core structures of many marketed drugs [103]. Chen et al., who first reported the preparation of universal convertible Rink-isocyanide resin **57** (cf. Scheme 11), used this support for the traceless synthesis of 3-acylamino imidazo[1,2-*a*]pyridines **458** (Scheme 77) [8]. Acylation with acid chlorides and spontaneous cleavage at 50 °C yielded the target acylated products **458**. Attempts to carry out base-mediated acylation or sulfonation were not successful.

### 10.3. Miscellaneous

**Scheme 78:**
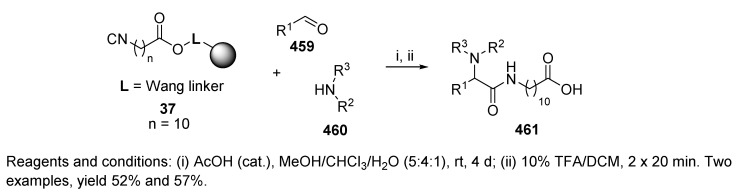
Synthesis of α-(dialkylamino)amides.

The first example in this section is the formation of α-(dialkylamino)amides **461** (Scheme 78), which are known to be formed in solution-phase synthesis when carboxylic acid is not present in the MCRs [7]. In the solid phase, addition of a catalytic amount of acetic acid was required in the reaction; otherwise, no product was formed. If other acidic catalysts or equivalents of acetic acid were used instead, a mixture of products (including Passerini-type adducts) was obtained.

**Scheme 79:**
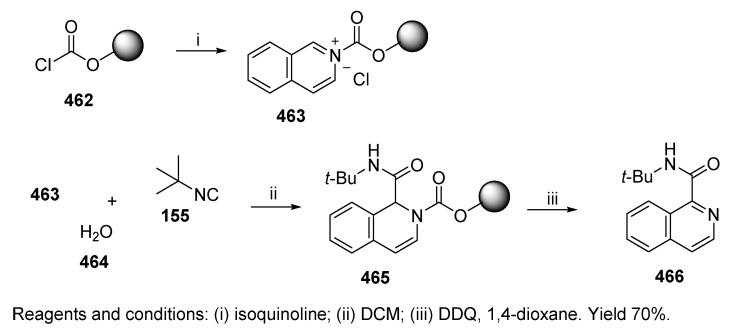
IMCR involving *N*-acylazinium ion.

Other IMCRs involved *N*-acylazinium salts as a source of iminium ions [9]. The reaction was initially studied in the solution phase. Treatment of azines (such as quinolines, isoquinolines, and phenanthridine) with activating agents (chloroformates, acid halides, or sulfonyl halides), isocyanide, and water, yielded 1,2-dihydroazine-1-carboxamides. In the solid phase, *N*-acyl isoquinoline ion **463** (Scheme 79) was reacted with *tert*-butyl isocyanide and water, and the target isoquinoline-1-carboxamide **466** was liberated by oxidative cleavage in 70% yield. Only one example was given.

## 11. Conclusions and Future Perspectives

In conclusion, U-4CC has become an established and robust synthetic method, as documented by numerous reports, including syntheses of sizable libraries for drug discovery. There are several avenues to fully exploit their potential, and they particularly include the following four areas of research, which are mostly focused on drug discovery:

(i) The advanced intermediates prepared by U-4CC with three or four diversity positions can be further modified by derivatization, which can lead to structurally complex chiral frameworks with three-dimensional architecture. In addition to the previously reported Diels-Alder reaction [3,96], the cyclic iminium–nucleophilic addition tandem reaction shows great potential [104].

(ii) DNA-encoded libraries represent a cutting-edge technology in drug discovery and are used by numerous pharmaceutical companies. The current restriction of the technology is the need to use mild reaction conditions compatible with DNA. Due to their mild reaction conditions, U-4CC open a new potential in this avenue [18].

(iii) Ugi reactions are already carried out under environmentally friendly reaction conditions. 2-MeTHF as a solvent is applied for SP [15]. In addition, the MCRs do not require the use of any additional reagents, and high atom economy and mild reaction conditions are additional attributes of sustainable organic synthesis [14].

(iv) The formation of a new asymmetric center, particularly in the synthesis of novel α-amino acids, is probably the most useful BB in the synthesis of novel structures. Chiral auxiliary has already been shown to enable direct enantio-enriched compounds [19,20].

IMCRs play an important role in organic synthesis, including the drug discovery process. High atom economy, high yields of products, mild reaction conditions, and no need for additional reagents or special laboratory equipment belong to the most indisputable highlights of these reactions. The solid-phase approach provides a huge advantage in the possibility of producing of chemical libraries in a short time with simple isolation of products that may markedly facilitate searching for new pharmacologically relevant molecules.
